# WWP1 gain-of-function drives developmental anoikis through TGFβ pathway during neurodevelopment

**DOI:** 10.1038/s41420-026-02977-4

**Published:** 2026-03-06

**Authors:** Ki Hurn So, Seungbok Lee, Jiayi Wong, Hyunsik Lee, Eun-Jin Yun, Se Song Jang, Hee-Jung Choi, Jong-Hee Chae, Seung Tae Baek

**Affiliations:** 1 Department of Life Sciences, Pohang University of Science and Technology (POSTECH), Pohang, 37673 Republic of Korea; 2 Department of Genomic Medicine, Seoul National University Hospital, Seoul, 03080 Republic of Korea; 3 Department of Pediatrics, Seoul National University College of Medicine, Seoul, 03080 Republic of Korea; 4 Department of Biological Sciences, Seoul National University, Seoul, 08826 Republic of Korea; 5 Present Address: Bio R&D Center, Samsung Biologics, Incheon, South Korea

**Keywords:** Cell death in the nervous system, Neurodevelopmental disorders

## Abstract

The E3 ubiquitin ligase WWP1 orchestrates multiple cellular functions, yet the neurodevelopmental role and pathological implications of its dysregulation remain poorly defined, in contrast to its established oncogenic effects. Here, we demonstrate that hyperactive WWP1 induces neurodevelopmental abnormalities characterized by impaired neuronal migration and caspase-dependent cell death in the developing mouse brain and human neural progenitor cell models. Mechanistically, WWP1 gain-of-function (GOF) mutation disrupts cell adhesion, leading to anoikis, detachment-induced cell death. Pathway-level screening identifies TGFβ1 ligand treatment to restore cell survival in both neural progenitor cultures and embryonic mouse brains. Conversely, TGFβ pathway inhibition phenocopies WWP1-induced apoptosis, establishing that WWP1 hyperactivity promotes cell death via TGFβ pathway downregulation. Transcriptomic profiling of the WWP1 GOF cellular models confirms the downregulation of cell adhesion and TGFβ signaling pathway signatures, highlighting the necessity of balanced WWP1 activity during neurodevelopment. In addition, we identified a de novo WWP1 variant in a patient with developmental and epileptic encephalopathy. Biochemical and in vivo functional analyses characterize the variant as GOF, supporting the clinical relevance of WWP1 dysregulation in neurodevelopmental disorders. Together, these findings reveal WWP1 as a critical regulator of neuronal survival and adhesion, with its dysregulation disrupting key developmental processes in the human brain.

## Introduction

The ubiquitin-proteasome system is essential for cellular protein homeostasis, enabling cells to respond to complex and dynamic demands while supporting vital functions [1–3]. Within this system, E3 ubiquitin ligases play a central role by transferring ubiquitin to substrate proteins, thereby regulating their stability, localization, and function across diverse biological processes [4–6]. WW domain-containing E3 ubiquitin protein ligase 1 (WWP1) is a member of the NEDD4 family of HECT-type ubiquitin E3 ligases containing a C2 domain and four tandem WW domains [7–9]. Similar to other NEDD4 family members, WWP1 modulates several signaling cascades such as TGFβ, PTEN/AKT, and EGFR [9–14]. Aberrant WWP1 activity and function has been implicated in multiple human diseases, ranging from cancer to infectious and neurological disorders [8, 9, 15–17]. Numerous cancer-associated WWP1 mutations have been shown to enhance its ligase activity by disrupting auto-inhibitory mechanisms, implicating gain-of-function (GOF) mutations in disease pathogenesis [18–21]. However, the relevance of WWP1 hyperactivation beyond oncogenesis remains relatively uncharacterized. WWP1 HECT-domain variants have been identified in patients with autism spectrum disorder and intellectual disability, suggesting potential roles in neurodevelopmental conditions through mechanisms that are yet to be elucidated [22]. Recent findings demonstrate that GOF mutations in another HECT-type E3 ligase, UBE3A, cause autistic neurodevelopmental abnormalities through mechanisms distinct from those observed in Angelman syndrome, which results from UBE3A loss-of-function, highlighting the importance of investigating the neurodevelopmental consequences of WWP1 hyperactivation [23, 24].

Proper cerebral cortex formation relies on precise molecular and cellular regulation during neurodevelopment [25–28]. Neuroepithelial cells develop into apical radial glia, which either self-replicate to maintain the neural progenitor pool or generate neurons that migrate into the cortical plate. Apical radial glia cells require precise regulation of adhesion molecules for their survival and differentiation, as disruption of cell adhesion frequently results in neurodevelopmental abnormalities [29–32]. However, the molecular links connecting ubiquitin-proteasome system pathways to neural progenitor adhesion and survival, particularly those involving WWP1, remain poorly understood.

In this study, we demonstrate that WWP1 hyperactivation during neurodevelopment induces detachment-induced cell death through suppression of the TGFβ signaling pathway. We further identify and functionally characterize a de novo WWP1 variant in a patient with developmental and epileptic encephalopathy, establishing WWP1 as a critical regulator of brain development and a potential pathogenic driver for associated neurodevelopmental disorders.

## Results

### Neurodevelopmental pathologies induced by WWP1 GOF

**Fig. 1:**
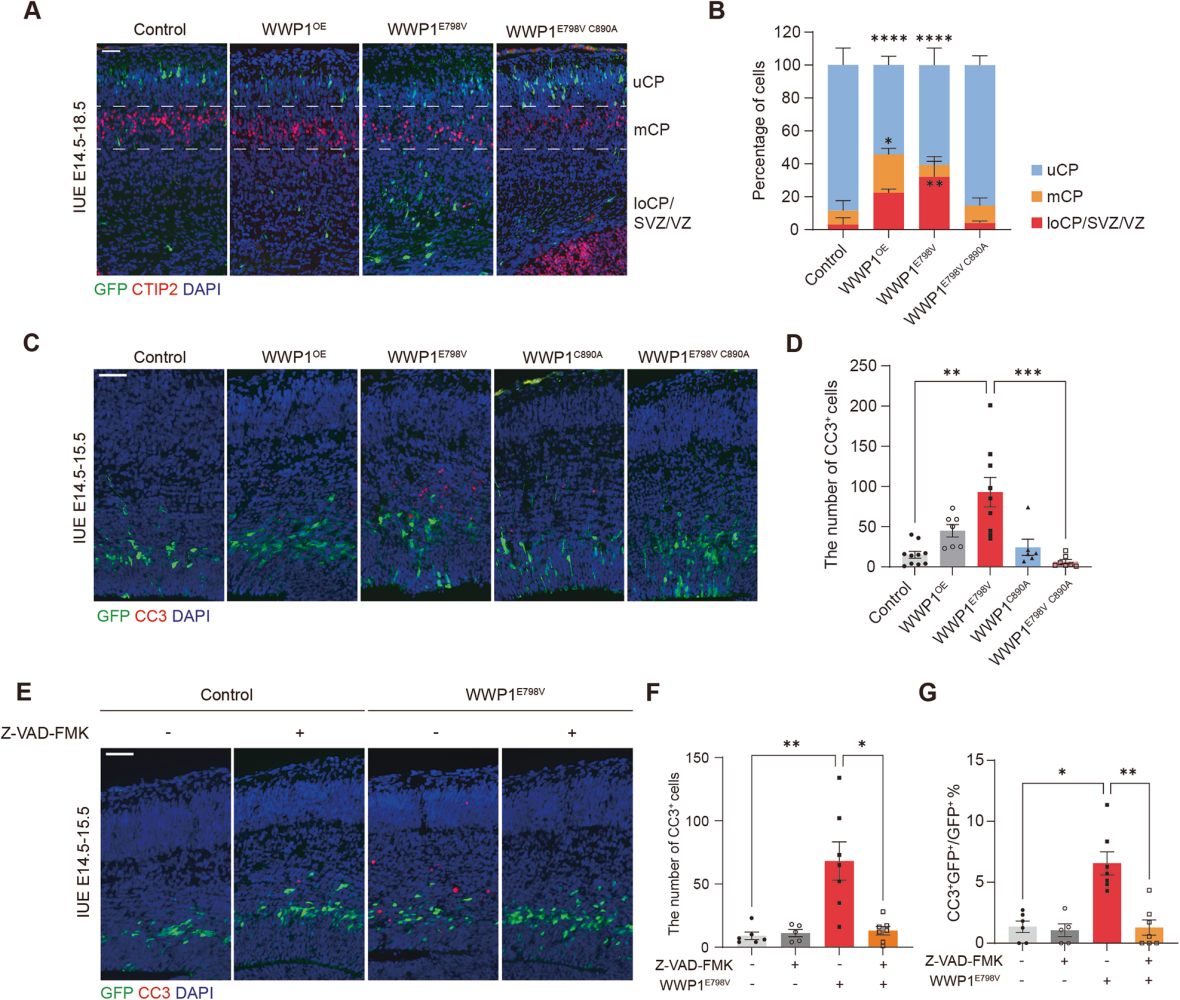
WWP1 GOF induced neurodevelopmental defects. **A** Representative images of E18.5 brain sections electroporated with control, WWP1^OE^, WWP1^E798V^, WWP1^E798V C890A^ constructs. Green, GFP; red, CTIP2; blue, DAPI. Dashed lines separated cortex zones into upper cortical plate (uCP), middle cortical plate (mCP), and lower cortical plate/subventricular zone/ventricular zone (loCP/SVZ/VZ). Scale bar, 50 μm. **B** Quantification of GFP^+^ cell distribution across cortical layers. *n* = 5 per group. Two-way ANOVA with Tukey’s post hoc test. *p < 0.05; **p < 0.01; ****p < 0.0001. **C** Representative images of E15.5 brain sections electroporated with control or WWP1 variants. Green, GFP; red, CC3; blue, DAPI. Scale bar, 50 μm. **D** Quantification of CC3^+^ cells in the electroporated region. Control, *n* = 10; WWP1^OE^, *n* = 7; WWP1^E798V^, *n* = 9; WWP1^C890A^, *n* = 6; WWP1^E798V C890A^, *n* = 7. Kruskal-Wallis test with Dunn’s post hoc test. **p < 0.01; ***p < 0.001. **E** Representative images of E15.5 brain sections electroporated with control or WWP1^E798V^ constructs administered with vehicle or Z-VAD-FMK. Green, GFP; red, CC3; blue, DAPI. Scale bar, 50 μm. **F, G**. Quantification of total CC3^+^ (**F**) and CC3^+^ among GFP^+^ cells (**G**) in the electroporated region. Control with vehicle, *n* = 6; control with Z-VAD-FMK, *n* = 5; WWP1^E798V^ with vehicle, *n* = 7; WWP1^E798V^ with Z-VAD-FMK, *n* = 7. Kruskal-Wallis test with Dunn’s post hoc test. *p < 0.05; **p < 0.01. Bar graphs indicate mean ± SEM.

Despite the established role in tumor progression, little is known about the broader biological impact of WWP1 hyperactivity, particularly within the WWP1-expressing fetal brain [33, 34]. To investigate the neurological effects of WWP1 GOF during neurodevelopment, we selected E798V, a hyperactive WWP1 variant previously identified in cancer research [18]. We characterized the effects of the WWP1 GOF by introducing vectors expressing green fluorescent protein (GFP) and WWP1 variants into the developing mouse cortex via *in utero* electroporation (IUE) at embryonic day (E)14.5. While the majority of control vector-electroporated cells migrated to the upper cortical plate (uCP), 46% of cells expressing wild-type WWP1 (hereafter referred to as WWP1^OE^) and 40% of cells expressing WWP1^E798V^ failed to reach the destined uCP and instead mislocalized in the middle or lower cortical plate (Fig. 1A, B). Notably, we observed a reduction in GFP^+^ cells in the WWP1^E798V^ group, suggesting potential defects in proliferation or cell survival (Fig. S1A).

To investigate the early neurodevelopmental impacts of WWP1 GOF, we analyzed electroporated brains at E15.5. WWP1^E798V^ expression did not affect proliferation, as indicated by comparable proportions of Ki67^+^ electroporated cells between the WWP1^E798V^ and control groups (Fig. S1B, C). However, WWP1^E798V^-electroporation caused a significant increase in apoptotic cells in the electroporated region, visualized by cleaved caspase 3 (CC3) staining, which was absent in both the non-targeted contralateral hemisphere and control vector-electroporation (Fig. 1C, D, and S1D). Notably, WWP1^OE^ exhibited a less severe phenotype, suggesting that cortical development is sensitive to WWP1 dosage and/or activity level.

To assess whether E3 ligase activity is required for WWP1 GOF-induced neurodevelopmental deficits, we utilized the WWP1^E798V^ construct containing an additional catalytically inactive C890A mutation [35, 36]. While expression of WWP1^C890A^ alone did not affect cell survival, the WWP1^E798V C890A^ double mutant-electroporation prevented both early apoptosis and migration defects, demonstrating that the neurodevelopmental pathologies, resembling that in cancer, are dependent on ligase activity (Fig. 1A–D) [18, 36].

Considering the widespread distribution of CC3^+^ cells beyond the electroporated population, suggesting potential non-cell autonomous effects, we tested whether inhibiting caspase activity could mitigate the observed phenotype. Intracerebroventricular administration of the pan-caspase inhibitor Z-VAD-FMK at E14.5 reduced total CC3^+^ cells and CC3^+^ electroporated cells, confirming that WWP1 GOF expression triggers caspase-dependent cell death (Fig. 1E–G).

To investigate the late-stage effects of WWP1 GOF, we analyzed the postnatal day (P) 7 brains electroporated at E14.5 with either empty vector or WWP1^E798V^. Unlike the migration defect observed in the embryonic stage, cells electroporated with WWP1^E798V^ reached the upper cortical layers, indicating that the migration defect was temporal (Fig. 1A and Fig. S1E). However, GFP intensity across the cortical plate was reduced compared to the control, which aligned with cell death at E15.5 (Fig. 1C–G and Fig. S1F). Furthermore, WWP1^E798V^-electroporated cells showed a significant decrease in SATB2 positivity, a marker of upper-layer neuronal fate, suggesting that WWP1 GOF exerted a lasting effect on postnatal cortical organization with cellular loss and altered cell fate specification (Fig. S1G).

WWP1, along with its homolog WWP2, has been reported to regulate neuronal polarization and cortical lamination in a *Wwp1*/2 double knockout mouse model [37]. Given their structural similarity and functional redundancy, we tested whether the WWP2 GOF variant (p.R841H) identified in a patient with herpes simplex encephalitis would recapitulate the acute cell death [9, 38]. Overexpression of WWP2^R841H^ in the developing cortex resulted in elevated apoptosis comparable to WWP1^E798V^, suggesting the functional similarity of WWP1 and WWP2 extends beyond oncogenesis into neurodevelopmental context (Fig. S1H, I).

These findings collectively demonstrate that hyperactive WWP1 promotes caspase-dependent apoptosis and migration defects through its E3 ligase function.

### Detachment-induced cell death by WWP1 GOF

**Fig. 2:**
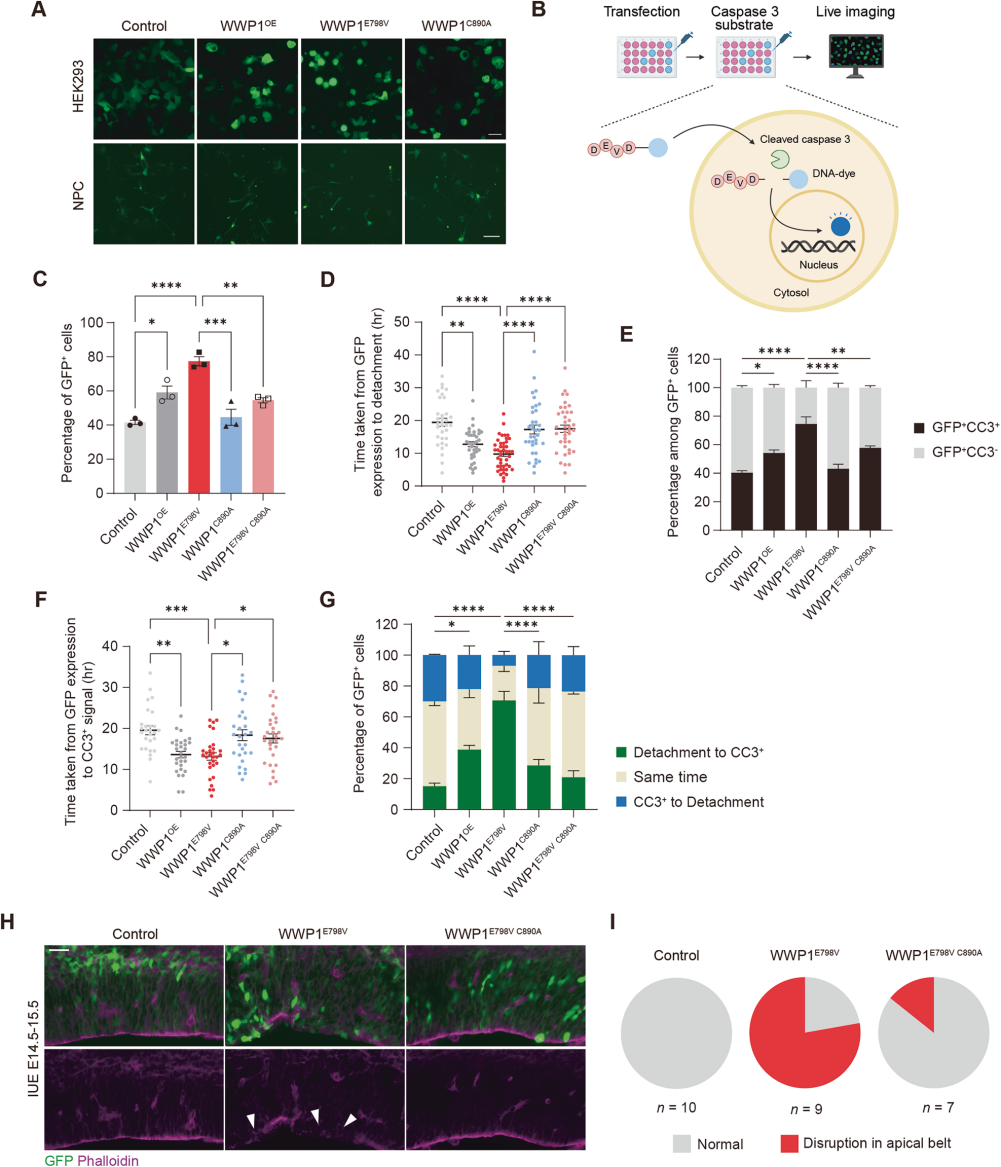
WWP GOF led to detachment-induced apoptosis. **A** Representative images of HEK293T and hNPCs transfected with control or WWP1 variant constructs co-expressing GFP. Scale bar, 50 μm. **B** Scheme of live imaging to monitor apoptosis events in hNPCs. The image is created with Biorender.com. Fluorogenic dye is activated upon cleavage by active caspase 3. **C** Quantification of the percentage of detached cells among GFP^+^ cells transfected with control or WWP1 variants. *n* = 3. One-way ANOVA with Tukey’s post hoc test. *p < 0.05; **p < 0.01; ***p < 0.001; ****p < 0.0001. **D** Quantification of time taken from GFP expression to detachment in hNPCs transfected with control or WWP1 variants. Control, *n* = 33; WWP1^OE^, *n* = 36; WWP1^E798V^, *n* = 44; WWP1^C890A^, *n* = 36; WWP1^E798V C890A^, *n* = 39. Kruskal-Wallis test with Dunn’s post hoc test. **p < 0.01; ****p < 0.0001. **E** Quantification of the percentage of GFP^+^CC3^+^ and GFP^+^CC3^-^ hNPCs transfected with control or WWP1 variants. *n* = 3. Two-way ANOVA with Tukey’s post hoc test. The significance of the GFP^+^CC3^+^ group is labeled. *p < 0.05; **p < 0.01; ****p < 0.0001. **F** Quantification of time taken from GFP expression to CC3 signal in hNPCs transfected with control or WWP1 variants. Control, *n* = 27; WWP1^OE^, *n* = 29; WWP1^E798V^, *n* = 30; WWP1^C890A^, *n* = 28; WWP1^E798V C890A^, *n* = 30. Kruskal-Wallis test with Dunn’s post hoc test. *p < 0.05; **p < 0.01; ***p < 0.001. **G** Quantification of the percentage of hNPCs transfected with control or WWP1 variants categorized by detachment and apoptosis sequence. *n* = 3. Two-way ANOVA with Tukey’s post hoc test. Significance of the detachment to CC3^+^ group is labeled. *p < 0.05; ****p < 0.0001. **H** Representative images of apical junction belt in E15.5 brain electroporated with control or WWP1 variants. Green, GFP; magenta, Phalloidin. White arrows indicate disruption in the apical belt. Scale bar, 50 μm. **I** Pie chart showing the ratio of disrupted apical belt in E15.5 brain electroporated with control or WWP1 variants. Control, *n* = 10; WWP1^E798V^, *n* = 9; WWP1^E798V C890A^, *n* = 7. Bar graphs indicate mean ± SEM.

To further investigate the cellular impact of WWP1 GOF mutation, we expressed WWP1 variants in human HEK293T cells and neural progenitor cells (NPCs). Both WWP1^OE^ and WWP1^E798V^ expression resulted in rounded cell morphology, with most cells floating in the media (Fig. 2A). Given that neuronal cells are particularly susceptible to detachment-induced cell death, known as anoikis, we performed live-cell imaging on NPCs transfected with WWP1 variants to monitor detachment dynamics (Fig. 2B) [39–42]. WWP1^E798V^-transfected cells showed a marked increase in detachment 36 h post-transfection, detaching more rapidly than those transfected with either the GFP vector or WWP1^C890A^ (Fig. 2C, D).

To further examine cell fate, we tracked cell survival using a CC3-dependent fluorescent dye to detect apoptotic events (Fig. 2B). WWP1^E798V^-expressing cells exhibited significantly elevated apoptosis rates with faster progression than controls (Fig. 2E, F). Analysis of the sequence of detachment and apoptosis in individual cells revealed that cells expressing WWP1^E798V^ typically detached prior to undergoing apoptosis (Fig. 2G, Movie S1, S2). Consistent with in vivo findings, WWP1^OE^-transfected NPCs showed a similar but less pronounced trend compared to WWP1^E798V^, while the WWP1^E798V C890A^-expressing NPCs displayed low levels of detachment and apoptosis comparable to the GFP vector-transfected controls, indicating that both gain-of-function and ligase activity are required for the detachment and subsequent cell death phenotype (Fig. 2C–G).

Previous research has shown that pathological detachment of NPCs, termed developmental anoikis, can trigger caspase-mediated apoptosis in the embryonic neocortex [32]. We therefore examined whether WWP1 GOF disrupts apical adhesion structures near the ventricle. Consistent with in vitro findings, phalloidin staining at E15.5 revealed marked disorganization of the apical adherens junction belt in regions electroporated with WWP1^E798V^ at E14.5. This disorganization was less pronounced in regions electroporated with the catalytically inactive WWP1^E798V C890A^ mutant (Fig. 2H, I).

Collectively, our findings demonstrate that WWP1 GOF expression leads to detachment-induced cell death in NPCs, paralleling the phenotype observed in mouse IUE experiments.

### Adhesion-related protein enrichment in WWP1 substrates

Given that the phenotypes induced by WWP1 hyperactivation are dependent on its E3 ligase activity, we explored potential WWP1 substrates that may be involved in the detachment-induced cell death caused by WWP1 GOF using the Ubibrowser 2.0 platform [43]. Among 30 known and 1523 predicted substrates, gene ontology (GO) analysis revealed significant enrichment of signal transduction, development, and cell adhesion-related terms (Fig. S2A, B). We further analyzed previously published mass spectrometry data, identifying WWP1-interacting proteins in a human intrahepatic cholangiocarcinoma cell line [44]. A total of 76 WWP1-interacting proteins were categorized into four clusters by STRING analysis, with one of the clusters significantly enriched in cellular junction-related functions (Fig. S2C, D). To assess the developmental relevance of these interactors, we re-analyzed single-cell transcriptomic data from the developing mouse cerebral cortex [25]. Nearly half (48.7%, 37 out of 76) of the WWP1-interacting proteins were expressed in Pax6^+^ apical progenitors and cycling glial cells, populations dependent on apical adhesion for survival and polarity maintenance (Fig. S2E, F). These results suggest that WWP1 may regulate a broad set of adhesion-associated proteins in neural progenitors, potentially explaining the detachment-induced cell death upon WWP1 hyperactivation.

### Involvement of TGFβ pathway in WWP1 GOF-induced cell death

**Fig. 3:**
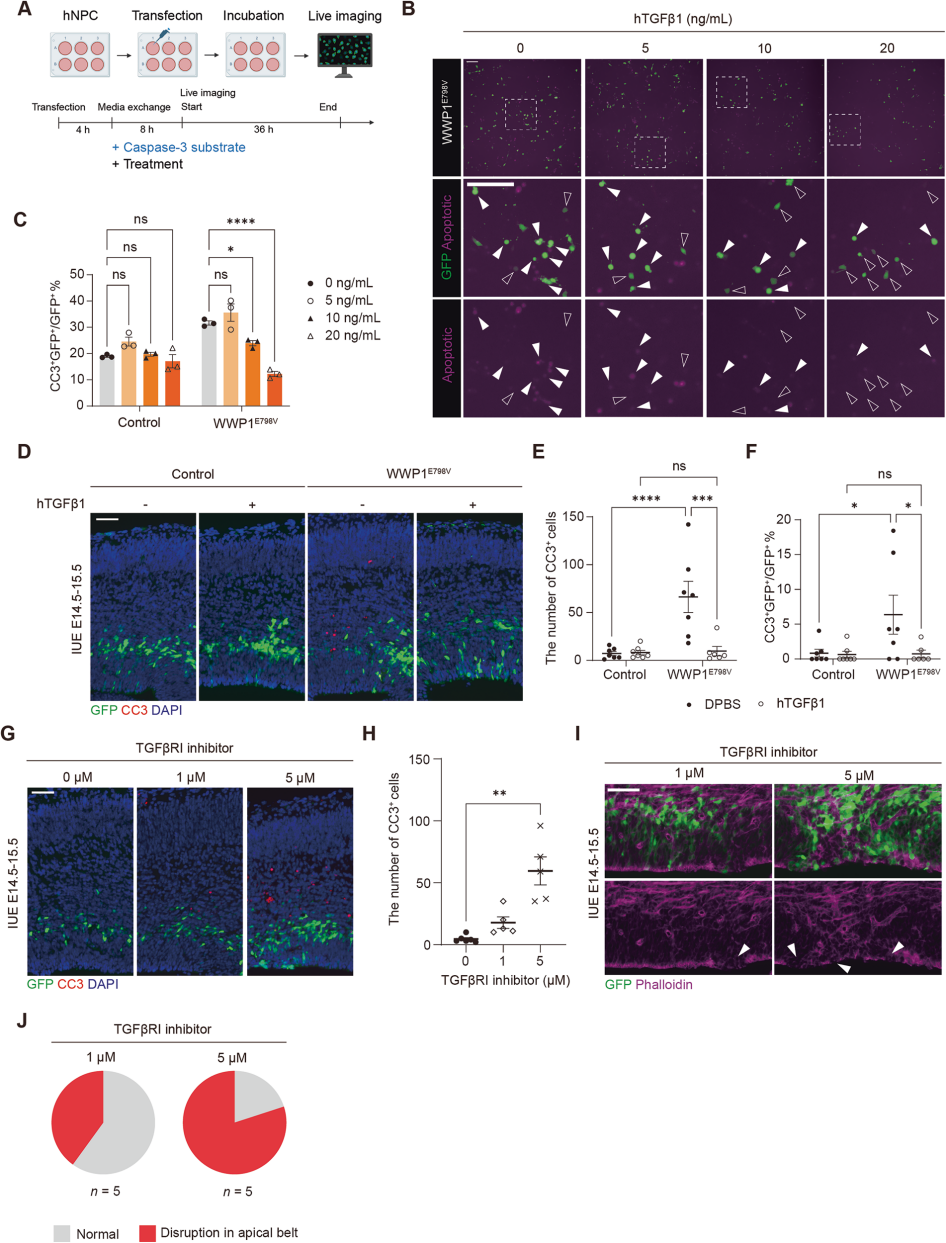
TGFβ pathway is involved in WWP1 GOF-induced cell death. **A** Scheme of live imaging to monitor pathway-oriented screening for apoptosis rescue in hNPCs. The image is created with Biorender.com. Caspase-3 substrate and treatment are administered 4 h after transfection. **B** Representative images of WWP1^E798V^-expressing hNPCs treated with different doses of hTGFβ1. Green, GFP; magenta, activated caspase-3 substrate signal. The inset with dashed lines was magnified for visualization. White arrows indicate GFP^+^ apoptotic cells, while blank arrows indicate GFP^+^ non-apoptotic cells. Scale bar, 200 μm. **C** Quantification of the percentage of apoptotic cells among GFP^+^ cells transfected with control or WWP1^E798V^. *n* = 3. Two-way ANOVA with Dunnett’s post hoc test. *p < 0.05; ****p < 0.0001; ns, not significant. **D** Representative images of E15.5 brain sections electroporated with control or WWP1^E798V^ constructs administered with vehicle or hTGFβ1. Green, GFP; red, CC3; blue, DAPI. Scale bar, 50 μm. Quantification of total CC3^+^ (**E**) and CC3^+^ among GFP^+^ cells (**F**) in the electroporated region. Control with vehicle, *n* = 7; control with hTGFβ1, *n* = 7; WWP1^E798V^ with vehicle, *n* = 7; WWP1^E798V^ with hTGFβ1, *n* = 6. Two-way ANOVA with uncorrected Fisher’s LSD post hoc test. ***p < 0.001; ****p < 0.0001; ns, not significant. **G** Representative images of E15.5 brain sections electroporated with GFP-expressing vector and administered with different doses of TGFβRI inhibitor. Green, GFP; red, CC3; blue, DAPI. Scale bar, 50 μm. **H** Quantification of total CC3^+^ in the electroporated region administered with TGFβRI inhibitor. 0 μM, *n* = 6; 1 μM, *n* = 5; 5 μM, *n* = 5. Kruskal-Wallis test with Dunn’s post hoc test. **p < 0.01. **I** Representative images of apical junction belt in E15.5 brain sections electroporated with GFP-expressing vector and administered with different doses of TGFβRI inhibitor. Green, GFP; magenta, Phalloidin. White arrows indicate disruption in the apical belt. Scale bar, 50 μm. **J** Pie chart showing the ratio of disrupted apical belt in E15.5 brain sections electroporated with GFP-expressing vector and administered with different doses of TGFβRI inhibitor. 1 μM, *n* = 5; 5 μM, *n* = 5. Bar graphs indicate mean ± SEM.

Since many WWP1 substrates, including PTEN, EGFR, and SMAD4, are involved in signaling pathway regulation, we conducted pathway-oriented screening to identify signaling mediators that might rescue WWP1 GOF-induced cell death. We treated WWP1^E798V^-transfected hNPCs with various pathway inhibitors or ligands and monitored cell survival using a CC3-dependent fluorescent dye (Fig. 3A). While most pathway inhibitors failed to reduce WWP1 GOF-induced cell death at non-toxic concentrations, recombinant human TGFβ1 (hTGFβ1) treatment significantly decreased cell death caused by WWP1^E798V^ in a dose-dependent manner (Fig. 3B, C, and S3A–C).

To validate the rescue effect in vivo, we delivered hTGFβ1 into developing mouse brain ventricles during IUE. Consistent with in vitro results, hTGFβ1 administration significantly reduced total CC3^+^ cells and CC3^+^ electroporated cells in WWP1^E798V^-electroporated brains to levels comparable to control vector-electroporated brains. These results further support involvement of the TGFβ pathway in non-cell-autonomous cell death triggered by WWP1 hyperactivation (Figs. 1C, D and 3D–F).

Proper regulation of the TGFβ pathway is essential during neurodevelopment as evidenced by increased neuronal cell death upon TGFβ1 loss [45–50]. Studies have revealed that core components of TGFβ pathway are expressed in radial glial cells in the prenatal mouse brain, with pathway activation occurring in migrating neurons within the subventricular zone, suggesting that TGFβ pathway dysregulation would be deleterious during neurodevelopment [51, 52]. To confirm the critical role of TGFβ pathway inhibition in WWP1 GOF-induced deficits, we intracerebroventricularly delivered the TGFβRI inhibitor, galunisertib, together with a GFP-expressing control vector at E14.5 [53]. TGFβRI inhibition phenocopied non-cell-autonomous cell death and disruption of the apical adherens junction belt at E15.5 in a dose-dependent manner, suggesting that TGFβ pathway inhibition is sufficient to induce developmental anoikis similar to WWP1 GOF (Fig. 3G–J).

These findings together demonstrate that the TGFβ pathway is required for neural progenitor survival, and that its inhibition promotes the detachment-triggered apoptosis by WWP1 GOF.

### Transcriptomic perturbation of cell adhesion and TGFβ pathway signatures in WWP1 GOF cells

**Fig. 4:**
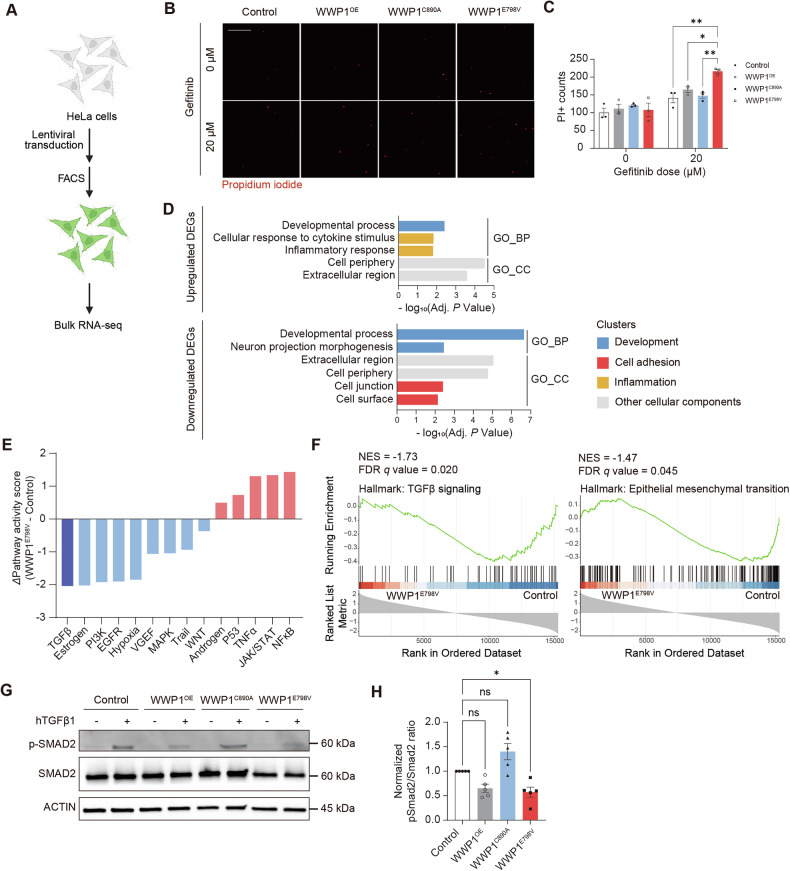
Transcriptomic analysis in WWP1 GOF cell line revealed attenuated TGFβ pathway activity. **A** Scheme of WWP1 variant-expressing cell line establishment and downstream analysis. The image is created with Biorender.com. **B** Representative images of dead cell population in WWP1 variants-expressing HeLa cell lines treated with gefitinib. Red, propidium iodide (PI). Scale bar, 200 μm. **C** Quantification of the PI^+^ counts in HeLa cell lines treated with gefitinib. *n* = 3 for each condition. Two-way ANOVA with Tukey’s post hoc test. *p < 0.05; **p < 0.01. **D** Functional GO enrichment analysis of upregulated and downregulated DEGs between WWP1^E798V^ and control cell lines. The bar color indicates the cluster categories of the GO term. BP, biological process; CC, cellular component. **E** Differential PROGENy pathway activity scores of WWP1^E798V^ relative to control. The pathway with the lowest value, TGFβ, is indicated by a deep blue color. **F** Gene set enrichment plots for TGFβ signaling and epithelial-mesenchymal transition. NES, normalized enrichment score. **G** Western blot analysis of phospho-SMAD2 and SMAD2 in WWP1 variant-expressing HeLa cell lines treated with vehicle or hTGFβ1 (10 ng/mL). ACTIN is used as the loading control. **H** Quantification of normalized pSMAD2 to SMAD2 protein expression ratio in WWP1 variant-expressing HeLa cell lines. *n* = 5. Kruskal-Wallis test with Dunn’s post hoc test. *p < 0.05; ns not significant. Bar graphs indicate mean ± SEM.

To uncover the molecular mechanisms underlying WWP1 hyperactivity, we established stable HeLa cell lines expressing WWP1 variants via lentiviral transduction, followed by fluorescence-based sorting (Fig. 4A). Although there were no significant changes in Ki67^+^ cell population by staining, cell cycle analysis revealed a decreased proportion of cells in G1 phase and an increased proportion of cells in S phase in WWP1^E798V^-expressing HeLa cells, consistent with its oncogenic relevance (Fig. S4A–D) [18]. WWP1 variant expression did not cause overt cell death during routine culture, indicated by no significant difference in CC3 staining and flow cytometry analysis (Fig. S4E–G). We hypothesized that cells with high expression of WWP1 GOF might have been negatively selected during the cell line establishment process due to heightened detachment susceptibility. Indeed, upon treatment with gefitinib, which imposes additional anoikis stress during cell seeding, WWP1^E798V^-expressing cells exhibited a significant increase in propidium iodide positivity, indicating enhanced sensitivity to detachment-induced apoptosis (Fig. 4B, C).

To characterize the transcriptional landscape associated with WWP1 hyperactivity, we performed bulk RNA sequencing on cells expressing WWP1^OE^ and WWP1^E798V^. WWP1 mRNA levels were confirmed to be at least fivefold higher than in control cells (Fig. S5A). Principal component analysis demonstrated distinct clustering based on WWP1 variant expression (Fig. S5B). Differential gene expression analysis revealed 149 and 241 differentially expressed genes (DEGs) in WWP1^OE^ and WWP1^E798V^ cells, respectively, with 105 overlapping genes, suggesting shared downstream effects of elevated WWP1 activity (Fig. S5C). Upregulated DEGs in WWP1^E798V^ showed enrichment in inflammation-related GO terms, including cellular response to cytokine stimulus (*CCL2*, *CCL7*, *CCL8*, and *CCL13*) (Fig. 4D and S5D). In contrast, downregulated DEGs, such as *MAP2*, *LAMC3*, and *IGF1*, were overrepresented in developmental processes such as neuron projection morphogenesis (Fig. 4D and S5E). Furthermore, among cellular component GO terms, downregulated DEGs (*ITGA11*, *NOTCH3*, and *CDH11*) were significantly enriched in cell junction, indicating transcriptomic dysregulation of cell adhesion pathways by WWP1 hyperactivation (Fig. 4D and S5F).

We next explored transcriptomic alterations in signaling pathway activity induced by WWP1 GOF. PROGENy pathway activity analysis revealed higher JAK-STAT, NFκB, and TNFα activity and lower Estrogen, PI3K, and TGFβ activity in WWP1^E798V^-expressing cells (Fig. 4E) [54]. Among dysregulated pathways, TGFβ pathway activity was the most attenuated in the WWP1^E798V^ group, indicated by the largest activity score difference between WWP1^E798V^-expressing and control cells. The TGFβ pathway is known to modulate cell adhesion via remodeling of the extracellular matrix and promoting epithelial-mesenchymal transition (EMT) [55–57]. Consistently, gene set enrichment analysis (GSEA) showed significant enrichment in TGFβ signaling and EMT hallmarks in the control group compared to the WWP1^E798V^ group (Fig. 4F). Indeed, WWP1^E798V^-expressing cells showed decreased SMAD2 phosphorylation upon hTGFβ1 treatment, indicating lower pathway responsiveness (Fig. 4G, H). Since previous cancer research has reported that WWP1 negatively regulates TGFβ signaling by targeting pathway components as substrates, we examined protein levels of core receptors and downstream effectors, including TGFβRI, TGFβRII, and SMAD3. However, no significant changes were observed either at the basal level or following TGFβ treatment (Fig. S6A) [18, 35, 58]. The limited alteration at the protein level further supports that cells with high WWP1 GOF expression may have been selectively lost during cell line establishment. Instead, *TGFBI*, one of the genes known to decrease upon TGFβ pathway inhibition, showed a decreased mRNA level in WWP1^E798V^-expressing cells by quantitative PCR (qPCR) validation, indicating attenuated TGFβ pathway activity (Fig. S6B) [59]. In line with this, hNPCs transfected with WWP1^E798V^ showed significantly reduced mRNA levels of downstream transcriptional targets of the TGFβ pathway, such as *TGFBI* and *PMEPA1* (Fig. S6C) [60–62]. In contrast, no interactions were detected between WWP1 and upstream components or the transcriptional modulator, such as TGFβRI, TGFβRII, and SMAD2 (Fig. S6D).

Taken together, our results revealed that WWP1 hyperactivation led to transcriptional downregulation of cell adhesion and TGFβ signaling pathways, which aligns with the developmental anoikis phenotype observed in both the mouse cortex and hNPC models.

### De novo WWP1 variant identified in a patient with a neurodevelopmental disorder

**Fig. 5:**
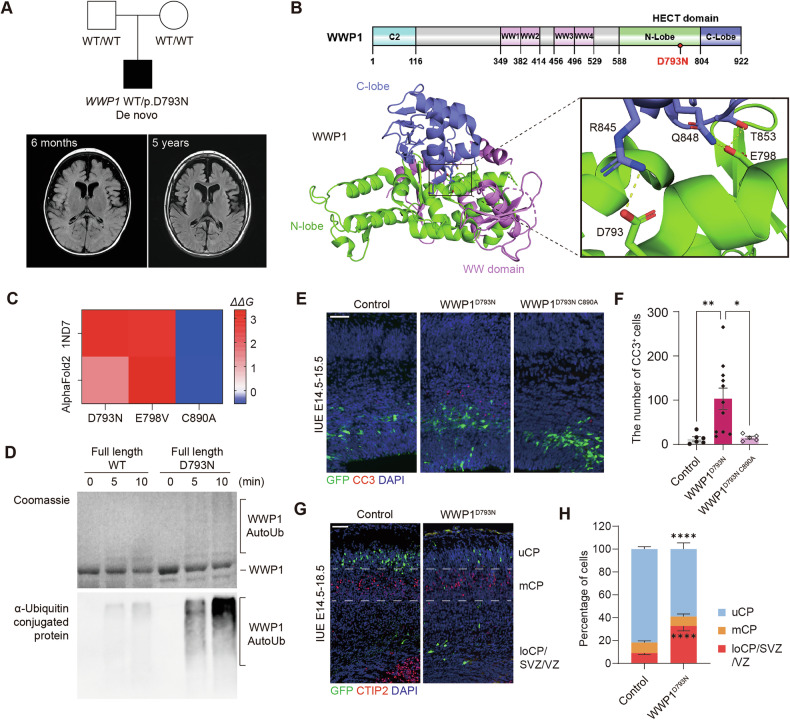
WWP1^D793N^ resulted in neurodevelopmental pathology comparable to WWP1 GOF. **A** Patient’s pedigree and brain MRI performed at 6 months and 5 years of age. The MRI image at 6 months of age showed subarachnoid space widening, particularly in the bilateral frontal regions and sylvian fissures, suggestive of diffuse brain atrophy. These findings showed mild progression on follow-up imaging at 5 years of age. **B** Domain architecture and structural model of WWP1 (PDB: 6J1X), highlighting the position of D793. Colors indicate distinct domains. The inset shows interactions of the D793 and E798 residues. **C** Perturbation modeling of WWP1 variants (D793N, E798V, C890A) using FoldX on both the resolved (PDB: 1ND7) and Alphafold2-predicted WWP1 structures. Color scale represents the predicted change in stability (*ΔΔG*). **D** In vitro autoubiquitination assay of full-length WWP1 wild-type and D793N variant. Representative Coomassie-stained gel and western blot images show levels of autoubiquitinated WWP1. **E** Representative images of E15.5 brain sections electroporated with control or WWP1^D793N^. Green, GFP; red, CC3; blue, DAPI. Scale bar, 50 μm. **F** Quantification of CC3^+^ cells in the electroporated region. Control, *n* = 6; WWP1^D793N^, *n* = 11; WWP1^D793N C890A^, *n* = 5. Kruskal-Wallis test with Dunn’s post hoc test. *p < 0.05; **p < 0.01. **G** Representative images of E18.5 brain sections electroporated with control and WWP1^D793N^ constructs. Green, GFP; red, CTIP2; blue, DAPI. Dashed lines separated cortex zones into uCP, mCP, and loCP/SVZ/VZ. Scale bar, 50 μm. **H** Quantification of GFP^+^ cell distribution across cortical layers. *n* = 5 per group. Two-way ANOVA with Šídák’s post hoc test. ****p < 0.0001. Bar graphs indicate mean ± SEM.

Despite the observed impact of WWP1 GOF mutation on neurodevelopment, its direct association with human neurodevelopmental disorders remained speculative. Exome sequencing in a cohort of patients with undiagnosed neurodevelopmental disorders identified a de novo heterozygous WWP1 variant (c.2377 G > A, D793N) in a patient presenting developmental and epileptic encephalopathy with diffuse brain atrophy (Fig. 5A). Trio-based whole-genome sequencing identified no additional pathogenic variants. Clinically, the patient developed Lennox-Gastaut syndrome with refractory seizures from early childhood, leading to profound developmental regression and a bedridden state.

Structural analysis of WWP1 showed that D793 is located in the N-lobe of the HECT domain, where it forms an electrostatic interaction with R845 in the C-lobe (Fig. 5B). Previous structural and mechanistic studies have suggested that interdomain flexibility is important for the catalytic activity of WWP1 E3 ligase [19, 63]. The D793N mutation disrupts this electrostatic interaction, likely enhancing interdomain flexibility and thereby promoting catalytic activity. A similar mechanism may apply to the E798V mutation, which is predicted to disrupt polar interactions with Q848 and T853, located in the C-lobe. Consistently, computational analysis of the D793N and E798V mutations by FoldX predicted an increase in *ΔΔ*G, indicative of a destabilization effect on the domain interface (Fig. 5C) [64]. In line with structural analysis, in vitro ubiquitination assays showed that WWP1^D793N^ exhibits increased auto-polyubiquitination compared to the wild-type counterpart, suggesting that D793N is a putative GOF mutation (Fig. 5D).

To functionally validate this variant, we introduced WWP1^D793N^ into the developing mouse brain and observed significant apoptosis and migration defects during the embryonic stage, comparable to WWP1^E798V^ (Fig. 5E–G). Elevated apoptosis was rescued by introducing a catalytic-dead mutation, indicating that WWP1^D793N^ exhibited GOF properties similar to WWP1^E798V^ (Fig. 5E, F). These results suggest that WWP1 GOF mutations may contribute to previously uncharacterized neurodevelopmental disorders.

## Discussion

Dysregulated E3 ligases have been reported to hijack the shared molecular mechanisms in neurodevelopmental diseases and cancer [26]. In line with this, our findings show that developmental anoikis induced by WWP1 hyperactivation resembles the role of WWP1 in cancer metastasis. In gastric and prostate cancers, WWP1 overexpression enhances cell migration and invasion via the PTEN/PI3K and TGFβ pathways, respectively [8]. In osteosarcoma, enhanced WWP1 expression increased metastasis-associated MMP-2 and MMP-9, while promoting EMT by E-cadherin downregulation and β-catenin upregulation [17, 65]. Convergence into loss of epithelial polarity and acquisition of migratory potential are accompanied by anoikis resistance in cancer cells [66–69]. In contrast, our results suggest that the same ligase activity, when disrupted in the early neurodevelopment, leads to premature detachment-induced apoptosis. Adherens junction proteins that support tumor invasion are also critical for NPC survival and migration [26, 70–72]. This functional divergence highlights the cellular context-dependent role of WWP1 in regulating cell adhesion and fate.

Our findings indicated a suppressed TGFβ pathway transcriptional activity by WWP1 GOF in neural contexts. Accumulating evidence has suggested the involvement of WWP1 in the negative regulation of the TGFβ pathway, either by triggering degradation of several pathway components, such as TGFβRI and SMAD4, or by inhibiting TGFβ-responsive transcriptional activities, including PAI-1 expression [7, 9]. In addition, WWP2, a homolog of WWP1, has been reported to regulate the transcriptional activity of SMAD2 via mediating nucleocytoplasmic shuttling [73]. Given that no direct interactions were observed between WWP1 and upstream components or the transcriptional modulator in hNPCs, WWP1 GOF may suppress TGFβ signaling through alternative mechanisms, such as regulation of transcription factors or other signaling pathways, in the context of neurodevelopmental processes, which warrant further investigation (Fig. S6C) [9, 74].

While our results demonstrated that TGFβ1 administration can rescue hyperactive WWP1-induced apoptosis, strategies directly targeting WWP1 may offer additional therapeutic avenues. WWP1 has emerged as a promising druggable target in related diseases, including cancer [9, 75, 76]. For instance, indole-3-carbinol, a natural inhibitor of WWP1 found in cruciferous vegetables, showed significant anticancer efficacy against *Myc*-driven prostate tumors, serving as a foundation for the further development of more potent therapeutics in other diseases [36, 77, 78]. The availability of WWP1-modulating drugs may offer opportunities in therapeutic repurposing to address WWP1-associated neurodevelopmental diseases.

Consistent with several HECT-domain variants identified in patients exhibiting autism spectrum disorder and intellectual disability, our characterization of a de novo WWP1 variant underscores the clinical significance of precise WWP1 regulation in neurodevelopmental disorders. Clinical features observed in the patient, including diffuse brain atrophy, align well with our experimental evidence that WWP1 gain-of-function mutations trigger anoikis, and the variant was not found in the Bravo (https://bravo.sph.umich.edu/), TogoVar, and our in-house databases [79]. However, in gnomAD, the same variant has been found with the allele frequency of 2.48×10^-6^, raising the question of whether the variant is truly pathogenic [80]. Nevertheless, our biochemical analysis revealed that the WWP1^D793N^ variant enhanced WWP1 autoubiquitination, and in utero electroporation of WWP1^D793N^ led to neurodevelopmental pathologies comparable to the known hyperactive WWP1^E798V^ variant, supporting the dysfunction of WWP1 by D793N variant. The absence of overt clinical symptoms in the population database carriers of the D793N mutation may reflect incomplete penetrance or the occurrence of compensatory mutations at other loci. Incomplete penetrance has been observed in several neurodevelopmental disorders, including *LGI1*-associated autosomal dominant epilepsy with auditory features and *SCN1A*-related Dravet syndrome [81, 82]. Additionally, protective genetic or epistatic interactions have been described across neurological conditions. For instance, two distinct *DNM2* mutations showing antagonistic effects resulted in phenotypic rescue when combined, and an individual with the *APOE3* R136S mutation showed delayed Alzheimer’s disease onset despite carrying a pathogenic *PSEN1* variant [83, 84]. In particular, all four individuals with the variant in gnomAD were of non-Finnish European ancestry, with no occurrences reported in other populations, raising the possibility that an ethnicity-specific variant in cis may attenuate the GOF effect. Additional patient reports and more in-depth genomic studies are needed to establish a robust genotype–phenotype correlation and unravel the possible genetic architecture underlying *WWP1*-related neurodevelopmental disorders.

Several limitations of our study remain. First, although we demonstrate that WWP1 hyperactivation leads to TGFβ pathway inhibition, the precise molecular targets or substrates of WWP1 that function upstream of the TGFβ pathway remain to be identified. Second, our in vivo analyses mainly focus on acute embryonic effects and a lasting postnatal impact of WWP1 GOF. Future studies would require improved animal models for the comprehensive evaluation of postnatal brain development and behavioral phenotypes relevant to patient symptoms. Overall, our findings establish WWP1 as a critical regulator of neurodevelopment and uncover the mechanism by which its hyperactivation compromises NPC adhesion and survival through the TGFβ signaling pathway suppression.

## Materials and methods

### Plasmids

Human WWP1 variants (wild-type, D793N, E798V, C890A, E798V/C890A) and WWP2 variants (wild-type, R841H) were generated by block PCR-based mutagenesis and sub-cloned into the pCAG-IRES-GFP expression vector. For lentiviral packaging, FLAG-tagged WWP1 constructs were subcloned into pLV-EF1a-MCS-IRES-GFP (VectorBuilder Inc., Chicago, IL, USA). pCMV-VSV-G (Addgene, #8454) and pCMV-delta 8.91 plasmids were used as packaging components. Wild-type and D793N variants of WWP1 were cloned into pET21d by PCR using NEBuilder HIFI DNA Assembly (New England Biolabs). All plasmids were verified by DNA sequencing, and primer sequences for mutagenesis are listed in Table S1.

### In utero electroporation and tissue processing

All animal housing, handling, and experimental protocols adhered to guidelines approved by the Pohang University of Science and Technology’s Institutional Animal Care and Use Committee (POSTECH-2019-0071; POSTECH-2020-0077). C57/BL6 mouse strains obtained from Hyochang Science (Daegu, South Korea) were used for all experiments, incorporating both sexes. Timed pregnancy was achieved by designating the presence of a vaginal plug as E0.5. Surgical procedures followed the methodology previously described [85]. In brief, pregnant dams at E14.5 were anesthetized using isoflurane (induction: 3%, surgery: 2%, Hana Pharm Corporation, #657801261). Endotoxin-free plasmids (1 μg/μL), mixed with 0.1% Fast Green (Sigma, #F7252), were injected into the lateral ventricle of each embryo. Four 50 ms pulses of 45 V were delivered at 500 ms intervals using an ECM 830 Square Wave Electroporation System (BTX™-Harvard Apparatus). A 5 mm Platinum Tweezertrode (BTX™-Harvard Apparatus, #45-0489) was used to target neural progenitors in the cortical ventricular zone. Embryos from each condition were screened post-isolation to ensure consistent tissue integrity, targeted region, and transfection efficiency.

Brain samples from P7 pups were obtained by decapitation, while pregnant dams were euthanized by cervical dislocation under isoflurane anesthesia. Harvested E15.5 or E18.5 embryonic brains were fixed with 4% paraformaldehyde (PFA) in PBS for 6 h (E15.5) or overnight (E18.5) at 4 °C, washed with PBS, and immersed in 30% sucrose in PBS. P7 brains were fixed overnight at 4 °C in 4% PFA in PBS, then cryoprotected by sequential overnight incubation in 15% and 30% sucrose in PBS. Brains were embedded in Optimal Cutting Temperature (OCT) compound (Tissue-Tek, #HIO-0051), snap-frozen at –80 °C, and cryosectioned at 12 μm.

### Immunostaining

For immunohistochemistry, dried sections were rinsed three times with PBS, permeabilized using 0.1% Triton X-100 in PBS, and blocked with CAS-Block histochemical reagent (Invitrogen, #008120). Sections were incubated with primary antibodies overnight at 4 °C, followed by Alexa Fluor-conjugated secondary antibodies for 30 min at room temperature. For phalloidin staining, sections were incubated with Alexa Fluor^TM^ 568-conjugated phalloidin for 1 h at room temperature. Nuclei were counterstained with DAPI (Invitrogen, #D1306), and sections were mounted using 80% glycerol in PBS.

For immunocytochemistry, the protocol closely resembled immunohistochemistry with slight adjustments. Cells were grown on coverslips within 24-well plates. Cells were fixed with 4% paraformaldehyde for 15 min, washed three times with 1X PBS, treated with 200 μL of 0.1% Triton X-100 in PBS for 15 min, and blocked with CAS-Block histochemical reagent for 1 h at room temperature. Coverslips were transferred onto glass slides and mounted using 80% glycerol in PBS. Primary antibodies used in this study were as follows: SATB2 (Abcam, #ab51502, 1:400), CTIP2 (Abcam, #ab18465, 1:500), Ki67 (Abcam, #ab15580, 1:500), and cleaved caspase 3 (Cell Signaling Technologies, #9661, 1:500).

### Chemicals and reagents

Recombinant human TGFβ1 (PeproTech, #100-21) stock was prepared in 10 mM citric acid. Z-VAD-FMK (20 μM, MedChemExpress, # HY-16658B), recombinant human TGFβ1 (5 μg/mL), or galunisertib (1 or 5 μM, MedChemExpress, #HY-13226) were administered with plasmids intracerebroventricularly during IUE. Gefitinib (Sigma, #SML1657) was dissolved in DMSO (Sigma, #D2650) for stock preparation.

### Human cell line culture

HEK293T, LentiX^TM^ HEK293T, and HeLa cell lines were maintained in DMEM medium supplemented with 10% fetal bovine serum (Corning, #35-015-CV), 4.5 g/L glucose, 2 mM L-glutamine, and 100 U/mL penicillin/streptomycin (Gibco, #15140148). Medium was refreshed every three days, and cells were subcultured using 0.25% Trypsin/EDTA (Gibco, #25200072) upon reaching 100% confluency. hNPCs derived from embryonic stem cells were generated as described previously [85]. hNPCs were grown on Matrigel (Corning, #354234)-coated plates with DMEM/F12 supplemented with 0.5X B27 (Gibco, #17504044), 0.5X N2 (Gibco, #17502001), antibiotics, and bFGF (20 ng/mL, Stemcell Technologies, #78003). Passaging of hNPCs was performed with Accutase (Merck, #SCR005) upon 100% confluency. All cell lines were incubated at 37°C with 5% CO_2_ and confirmed to be mycoplasma-negative through regular PCR-based assays.

### Transfection and lentivirus transduction

Transfection was conducted using Lipofectamine 3000 (Invitrogen, #L3000015) and Opti-MEM medium (Gibco, #31985070) according to the manufacturer’s instructions. For lentivirus generation, LentiX^TM^ HEK293T cells were co-transfected with the pLV-EF1a-FLAG-WWP1-IRES-GFP lentiviral constructs and packaging plasmids using Lipofectamine 3000. Lentivirus-containing DMEM medium was collected at 48 and 72 h and purified through 0.45 μm filter (Pall Corporation, #4614). HeLa cells were infected with the lentivirus-containing medium for 24 h. Following a 72-h post-infection period, GFP^+^ HeLa cells were isolated using the Moflo Astrios flow cytometer (Beckman Coulter), with GFP^-^ cells serving as gating controls.

### Live Imaging and cell viability assays

Time-lapse live imaging was performed on a Nikon Ti2E microscope maintained at 37°C in 5% CO_2_. hNPCs were maintained in phenol red-free DMEM/F12 medium (Gibco, #11039021) supplemented with 0.5X B27, 0.5X N2, antibiotics, and bFGF. NucView® caspase-3 substrates (Biotium, #10405) were employed to visualize cells undergoing apoptosis following the manufacturer’s protocol. Cell detachment and apoptosis were tracked manually.

For signaling pathway screening, hNPCs were treated with various concentrations of hTGFβ1, galunisertib, LGK974, SB202190, miradametinib, tofacitinib, and LY2944002. Non-cytotoxic dosing conditions were determined by the cell viability using Propidium Iodide/RNase staining solution (Cell Signaling Technology, #4087). Non-toxic concentrations of treatments were added together with NucView® caspase-3 substrates upon live imaging. To assess the anoikis vulnerability of WWP1-stable HeLa cell lines, cells were seeded with 20 μM gefitinib. Cell viability was quantified using Propidium Iodide/RNase staining 48 h after seeding.

### Flow cytometry analysis

For cell cycle analysis, cells were collected at 60% confluence with 0.25% trypsin-EDTA and then washed twice with 1X PBS. The cell pellet was resuspended in 500 μL of 1X PBS, followed by gradual addition of 5 mL 70% ethanol dropwise along the tube wall. Ethanol-fixed cells were maintained at 4 °C until flow cytometric analysis. Fixed cells were washed with 3 mL 1X PBS and resuspended in 500 μL Propidium Iodide/RNase staining solution, incubated in the dark at room temperature for 15 min, and underwent flow cytometry analysis.

Apoptosis level was evaluated using the Annexin V-CF Blue/7-Aminoactinomycin D (7-AAD) Apoptosis Detection Kit (ab214663, Abcam) according to the manufacturer’s protocols. Briefly, adherent cells were gently detached using 0.25% trypsin-EDTA and washed twice in PBS at room temperature. Cells were then resuspended in 1X Binding Buffer Solution and treated with 5 μL Annexin V-CF Blue conjugate plus 5 μL 7-AAD staining solution per 100 μL cell suspension. Following 15-min incubation at room temperature in the dark, 400 μL of 1X Binding Buffer Solution was added.

All analyses were performed using CytoFLEX S (Beckman Coulter) with proper gating parameters and a minimum of 100,000 cells analyzed.

### RNA Sequencing and quantitative reverse-transcription PCR

Total RNA was extracted using the Quick RNA mini-prep kit (Zymo Research, #R1054). The library was prepared using the TruSeq stranded mRNA LT sample prep kit (Illumina) and sequenced on the HiSeq2000 platform. cDNA was synthesized using LunaScript reverse transcriptase (NEB, #E3010L) or ImProm-II™ Reverse Transcriptase (Promega, #A3802). qPCR experiments were conducted with Luna Universal SYBR reagent (NEB, #M3003L) on a MIC qPCR Cycler (BMS). Primer sequences were designed using the NCBI primer blast tool. *RPLP0* served as the reference gene for normalization. All primer sequences utilized are provided in Table S1.

### Transcriptomic analysis

Sequencing read quality was assessed using FastQC, followed by adapter trimming with Trimmomatic. Read alignment to the GRCh38_NCBI_109 reference genome was performed using Bowtie2. StringTie was subsequently employed to assemble aligned reads into recognized genes or transcripts and to compute read counts, FPKM, and TPM values. Differential gene expression analysis was conducted using the DESeq2 R package, excluding genes with total read counts below 13. DEGs were defined as genes with absolute fold changes greater than 2 and adjusted *P* values less than 0.05 between experimental conditions. GO enrichment analysis was conducted using g:profiler online tool. Heatmap visualization utilized *Z* scores derived from DESeq2’s variance stabilizing transformation function. The PROGENy method in R was applied to calculate signaling pathway activity [54]. Gene set enrichment analysis (GSEA) was executed using the GSEA R package [86]. WWP1 substrate information (known and predicted) was retrieved from the Ubibrowser 2.0 web-based platform [43]. Protein-protein interaction networks were displayed using the STRING online platform (https://string-db.org/). Single-cell RNA sequencing data from developing mouse cortex (GSE153164) were utilized to assess and visualize the expression patterns of WWP1-interacting proteins [25, 44].

### Protein extraction, immunoprecipitation, and Western blot analysis

Cells were gently rinsed with 1X DPBS supplemented with Ca^2+^ and Mg^2+^ before collection. RIPA lysis buffer (Rockland, #RKMB-030-0050) supplemented with phosphatase and protease inhibitors (Quartett, #QTPPI1041 and #QTPPI1015) was applied to the cells and incubated on a rocker at 12 rpm for 15 min for cell lysis. Proteins were collected using a cell scraper and preserved at –80 °C. Protein quantification was performed using Pierce™ BCA Protein Assay Kits (Thermo Scientific™, #23225) with protein standards. Protein samples were incubated in a shaking incubator at 37°C for 30 min at 150 rpm, and absorbance measurements were taken using a microplate reader (Tecan, Infinite® 200 PRO).

For immunoprecipitation, 100 μg of cell lysates were incubated with IgG (Cell Signaling Technologies, #2729) or FLAG antibody (Cell Signaling Technologies, #14793) in PBST (1X PBS containing 0.1% Tween-20) overnight with rotation at 4°C, and precipitated with Pierce Protein A/G Magnetic Beads (Thermo Scientific™, #88802) for 4 h with rotation at 4 °C. The immunocomplexes were subjected to Western blot analysis after gentle washing three times with PBST and elution with SDS-PAGE loading buffer.

Protein was separated using 4-15% Mini-PROTEAN precast gels (Bio-Rad, #4561086), transferred to nitrocellulose membranes (Bio-Rad, #1704159), and blocked with 5% non-fat dry milk (w/v) in PBST for 1 h at room temperature, and incubated overnight at 4 °C with primary antibodies including: ACTIN (Cell Signaling Technologies, #4970, 1:8000), SMAD2 (Cell Signaling Technologies, #5339, 1:1000), phospho-SMAD2 (Cell Signaling Technologies, #18338, 1:1000), TGFβR1 (Invitrogen, #AHO1552, 1:1000), TGFβRII (Invitrogen, #PA5-35076, 1:1000), SMAD3 (Invitrogen, #51-1500, 1:1000), phospho-SMAD3 (Invitrogen, #44-246 G, 1:1000), WWP1 (Abcam, #ab104440, 1:1000), FLAG (Cell Signaling Technologies, #8146, 1:1000). Following incubation with appropriate horseradish peroxidase-conjugated secondary antibodies (Promega, #W401B and #W402B), proteins were visualized using ECL substrate (Enzynomics, #EOE001S). Chemiluminescent signals were captured with the LAS4000 imaging system (GE Lifescience).

### Genetic analysis of an undiagnosed patient

The patient was recruited from the Rare Disease Center cohort at Seoul National University Hospital, Seoul, South Korea. Proper informed consent was secured from the participants, including permission to publish any photographic content when relevant. The research was approved by the Institutional Review Boards of Seoul National University Hospital (IRB no. SNUH 2204-112-1317) and conducted in accordance with the Helsinki Declaration guidelines.

Genomic DNA was isolated from blood samples collected from family trios (affected individual and both parents) and processed through clinical exome and genome sequencing using the Illumina platform following established protocols. The raw sequencing data were aligned to the hg38 human reference genome and analyzed using the Genome Analysis Toolkit (GATK) pipeline [87]. The *WWP1* c.2377 G > A variant (p.D793N) was determined to be the primary candidate mutation in this patient.

### In silico analysis of mutation effects

The WWP1 protein structure was obtained from the Protein Data Bank (PDB: 6J1X). Three-dimensional structural representations were generated and analyzed using PyMOL software (version 2.5.5; PyMOL Molecular Graphics System). The impact of mutations on the WWP1 crystal structure (PDB: 1ND7) and computationally predicted full-length protein structure (AF-Q0H0M0-F1) was evaluated via the FoldX5 software BuildModel function by calculating changes in *ΔΔ*G values.

### Protein expression and purification

For protein expression, pET21d plasmids were transformed into Escherichia coli Rosetta (DE3) cells and grown in 800 mL LB at 37 °C to an OD600 of ~0.6, at which point 0.2 mM IPTG was added and cultures were shaken for 16 h at 20 °C. Cells were harvested by centrifugation, resuspended in 20 mM Tris-HCl (pH 8.0) and 500 mM NaCl, and then stored at –80 °C.

Thawed cell pellets were treated with DNase I and 1 mM PMSF and lysed using an EmulsiFlex C3 homogenizer. After centrifugation, the clarified lysate was applied to Ni‑NTA agarose resin (Qiagen) pre-equilibrated in 20 mM Tris-HCl (pH 8.0), 500 mM NaCl, 1 mM DTT, and 20 mM imidazole, washed with ten column volumes of the same buffer, and eluted with buffer containing 300 mM imidazole. The eluate was then loaded onto a Mono Q anion exchange column on an ÄKTA FPLC system and eluted with a linear gradient of NaCl in 20 mM Tris-HCl (pH 8.0), before final polishing by size‑exclusion chromatography on a Superdex 200 Increase 10/300 GL column equilibrated in 20 mM Tris-HCl (pH 8.0) and 150 mM NaCl.

### In vitro autoubiquitination assay

Autoubiquitination reactions (50 µL) contained 100 nM UBE1, 1 µM UBE2L3 (UBCH7), 1 µM WWP1, and 10 µM ubiquitin in assay buffer composed of 50 mM Tris-HCl (pH 7.5), 5 mM MgCl2, 50 mM NaCl, 1 mM DTT, and 5 mM ATP. Reactions were incubated at 30 °C for 5 or 10 min, and subsequently quenched with SDS sample buffer. Proteins were resolved by SDS-PAGE on a 12% acrylamide/bis-acrylamide gel and analyzed by Coomassie Blue staining or Western blotting using an anti-ubiquitinylated proteins antibody (Sigma-Aldrich, #04-263, 1:2500).

### Imaging and quantification

Immunofluorescence-stained specimens were captured using either Nikon Eclipse Ts2R or Nikon Ti2E fluorescence microscopes. High-resolution confocal imaging was performed with the Olympus FV3000 confocal microscope. Live cell imaging was conducted using the Nikon Ti2E fluorescence microscope, with three randomly chosen fields per well. Images were processed using ImageJ, Adobe Photoshop CS6, and Illustrator CS6 software.

### Statistical and data analysis

This study did not employ pre-calculated sample sizes or power analyses. Data acquisition continued without predetermined endpoints, and all obtained data were incorporated into the final analysis. Each experiment was replicated at least three times with reproducible outcomes and contained a minimum of three biological replicates unless specified otherwise. All genotype assessment, treatment evaluation, and cell death quantification were performed by researchers blinded to experimental conditions.

Statistical analyses were conducted using R version 4.2.2, GraphPad Prism 10 (Graphpad Software, San Diego, CA), and MS Excel (Microsoft, Redmond, WA). For non-normally distributed data, the Kruskal-Wallis test was employed for multi-group comparisons. When data exhibited normal distribution but violated variance homogeneity assumptions, Brown-Forsythe and Welch’s ANOVA were utilized. In other cases, one-way or two-way ANOVA was applied to determine *p* values. For two-group comparisons with non-normal data distribution, the Mann-Whitney U test was used, while Student’s *t* test or Welch’s t test was selected based on variance homogeneity requirements. Multiple comparison corrections were applied using Tukey’s, Dunn’s, or Dunnett’s methods as appropriate. Results are expressed as mean ± standard error of the mean. Statistical significance was defined as *p* < 0.05, with significance levels denoted as *p* < 0.05 (*), < 0.01 (**), < 0.001 (***), or < 0.0001 (****) in data visualizations. Details regarding biological replicates, experimental procedures, and statistical methods are provided in the Figure legends.

## Supplementary information

Supplementary Information

Original Western Blot image

Movie S1

Movie S2

## Data Availability

The raw RNA-seq data have been deposited at SRA (PRJNA1292288) and are publicly available as of the date of publication. All data reported in this paper will be shared by the lead contact upon request.

## References

[1] Komander D, Rape M. The ubiquitin code. Annu Rev Biochem. 2012;81:203–29. 22524316 10.1146/annurev-biochem-060310-170328

[2] Sommer T, Wolf DH. The ubiquitin-proteasome-system. Biochim Biophys Acta. 2014;1843:1. 24055503 10.1016/j.bbamcr.2013.09.009

[3] Clague MJ, Urbe S, Komander D. Breaking the chains: deubiquitylating enzyme specificity begets function. Nat Rev Mol Cell Biol. 2019;20:338–52. 30733604 10.1038/s41580-019-0099-1

[4] Berndsen CE, Wolberger C. New insights into ubiquitin E3 ligase mechanism. Nat Struct Mol Biol. 2014;21:301–7. 24699078 10.1038/nsmb.2780

[5] Zheng N, Shabek N. Ubiquitin ligases: structure, function, and regulation. Annu Rev Biochem. 2017;86:129–57. 28375744 10.1146/annurev-biochem-060815-014922

[6] Lescouzeres L, Bomont P. E3 ubiquitin ligases in neurological diseases: focus on gigaxonin and autophagy. Front Physiol. 2020;11:1022. 33192535 10.3389/fphys.2020.01022 PMC7642974

[7] Zhi X, Chen C. WWP1: a versatile ubiquitin E3 ligase in signaling and diseases. Cell Mol Life Sci. 2012;69:1425–34. 22051607 10.1007/s00018-011-0871-7 PMC11114891

[8] Hu X, Yu J, Lin Z, Feng R, Wang ZW, Chen G. The emerging role of WWP1 in cancer development and progression. Cell Death Discov. 2021;7:163. 34226507 10.1038/s41420-021-00532-x PMC8257788

[9] Behera A, Reddy ABM. WWP1 E3 ligase at the crossroads of health and disease. Cell Death Dis. 2023;14:853. 38129384 10.1038/s41419-023-06380-0 PMC10739765

[10] Kato K, Miya F, Hori I, Ieda D, Ohashi K, Negishi Y, et al. A novel missense mutation in the HECT domain of NEDD4L identified in a girl with periventricular nodular heterotopia, polymicrogyria and cleft palate. J Hum Genet. 2017;62:861–3. 28515470 10.1038/jhg.2017.53

[11] Broix L, Jagline H, Ivanova E, Schmucker S, Drouot N, Clayton-Smith J, et al. Mutations in the HECT domain of NEDD4L lead to AKT-mTOR pathway deregulation and cause periventricular nodular heterotopia. Nat Genet. 2016;48:1349–58. 27694961 10.1038/ng.3676 PMC5086093

[12] Li K, Niu Y, Yuan Y, Qiu J, Shi Y, Zhong C, et al. Insufficient ablation induces E3-ligase Nedd4 to promote hepatocellular carcinoma progression by tuning TGF-beta signaling. Oncogene. 2022;41:3197–209. 35501461 10.1038/s41388-022-02334-6

[13] Komuro A, Imamura T, Saitoh M, Yoshida Y, Yamori T, Miyazono K, et al. Negative regulation of transforming growth factor-beta (TGF-beta) signaling by WW domain-containing protein 1 (WWP1). Oncogene. 2004;23:6914–23. 15221015 10.1038/sj.onc.1207885

[14] Inoue Y, Imamura T. Regulation of TGF-beta family signaling by E3 ubiquitin ligases. Cancer Sci. 2008;99:2107–12. 18808420 10.1111/j.1349-7006.2008.00925.x PMC11158544

[15] French ME, Klosowiak JL, Aslanian A, Reed SI, Yates JR 3rd, Hunter T. Mechanism of ubiquitin chain synthesis employed by a HECT domain ubiquitin ligase. J Biol Chem. 2017;292:10398–413. 28461335 10.1074/jbc.M117.789479 PMC5481553

[16] Haouari S, Vourc’h P, Jeanne M, Marouillat S, Veyrat-Durebex C, Lanznaster D, et al. The roles of NEDD4 subfamily of HECT E3 ubiquitin ligases in neurodevelopment and neurodegeneration. Int J Mol Sci. 2022;23:3882. 10.3390/ijms23073882 PMC8999422 35409239

[17] Lei J, Chen J, Yu W, Wu Q, Jing S, Tang Y, et al. Portrait of WWP1: the current state in human cancer. Front Cell Dev Biol. 2024;12:1516613. 39949609 10.3389/fcell.2024.1516613 PMC11821962

[18] Courivaud T, Ferrand N, Elkhattouti A, Kumar S, Levy L, Ferrigno O, et al. Functional characterization of a WWP1/Tiul1 tumor-derived mutant reveals a paradigm of its constitutive activation in human cancer. J Biol Chem. 2015;290:21007–18. 26152726 10.1074/jbc.M115.642314 PMC4543659

[19] Wang Z, Liu Z, Chen X, Li J, Yao W, Huang S, et al. A multi-lock inhibitory mechanism for fine-tuning enzyme activities of the HECT family E3 ligases. Nat Commun. 2019;10:3162. 31320636 10.1038/s41467-019-11224-7 PMC6639328

[20] Lee YR, Yehia L, Kishikawa T, Ni Y, Leach B, Zhang J, et al. WWP1 gain-of-function inactivation of PTEN in cancer predisposition. N Engl J Med. 2020;382:2103–16. 32459922 10.1056/NEJMoa1914919 PMC7839065

[21] Jiang H, Dempsey DR, Cole PA. Ubiquitin ligase activities of WWP1 germline variants K740N and N745S. Biochemistry. 2021;60:357–64. 33470109 10.1021/acs.biochem.0c00869 PMC8262107

[22] Novelli G, Novelli A, Borgiani P, Cocciadiferro D, Biancolella M, Agolini E, et al. WWP1 germline variants are associated with normocephalic autism spectrum disorder. Cell Death Dis. 2020;11:529. 32699206 10.1038/s41419-020-2681-z PMC7376150

[23] Weston KP, Gao X, Zhao J, Kim KS, Maloney SE, Gotoff J, et al. Identification of disease-linked hyperactivating mutations in UBE3A through large-scale functional variant analysis. Nat Commun. 2021;12:6809. 34815418 10.1038/s41467-021-27156-0 PMC8635412

[24] Xing L, Simon JM, Ptacek TS, Yi JJ, Loo L, Mao H, et al. Autism-linked UBE3A gain-of-function mutation causes interneuron and behavioral phenotypes when inherited maternally or paternally in mice. Cell Rep. 2023;42:112706. 37389991 10.1016/j.celrep.2023.112706 PMC10530456

[25] Di Bella DJ, Habibi E, Stickels RR, Scalia G, Brown J, Yadollahpour P, et al. Molecular logic of cellular diversification in the mouse cerebral cortex. Nature. 2021;595:554–9. 34163074 10.1038/s41586-021-03670-5 PMC9006333

[26] Cruz Walma DA, Chen Z, Bullock AN, Yamada KM. Ubiquitin ligases: guardians of mammalian development. Nat Rev Mol Cell Biol. 2022;23:350–67. 35079164 10.1038/s41580-021-00448-5

[27] Zhou Y, Song H, Ming GL. Genetics of human brain development. Nat Rev Genet. 2024;25:26–45. 37507490 10.1038/s41576-023-00626-5 PMC10926850

[28] Wang L, Wang C, Moriano JA, Chen S, Zuo G, Cebrian-Silla A, et al. Molecular and cellular dynamics of the developing human neocortex. Nature. 2025;647:169–78. 10.1038/s41586-024-08351-7 PMC12589127 39779846

[29] Winograd-Katz SE, Fassler R, Geiger B, Legate KR. The integrin adhesome: from genes and proteins to human disease. Nat Rev Mol Cell Biol. 2014;15:273–88. 24651544 10.1038/nrm3769

[30] Schmid MT, Weinandy F, Wilsch-Brauninger M, Huttner WB, Cappello S, Gotz M. The role of alpha-E-catenin in cerebral cortex development: radial glia specific effect on neuronal migration. Front Cell Neurosci. 2014;8:215. 25147501 10.3389/fncel.2014.00215 PMC4124588

[31] Buchsbaum IY, Kielkowski P, Giorgio G, O’Neill AC, Di Giaimo R, Kyrousi C, et al. ECE2 regulates neurogenesis and neuronal migration during human cortical development. EMBO Rep. 2020;21:e48204. 32207244 10.15252/embr.201948204 PMC7202216

[32] Laszlo ZI, Lele Z, Zoldi M, Miczan V, Mogor F, Simon GM, et al. ABHD4-dependent developmental anoikis safeguards the embryonic brain. Nat Commun. 2020;11:4363. 32868797 10.1038/s41467-020-18175-4 PMC7459116

[33] Wood JD, Yuan J, Margolis RL, Colomer V, Duan K, Kushi J, et al. Atrophin-1, the DRPLA gene product, interacts with two families of WW domain-containing proteins. Mol Cell Neurosci. 1998;11:149–60. 9647693 10.1006/mcne.1998.0677

[34] Flasza M, Gorman P, Roylance R, Canfield AE, Baron M. Alternative splicing determines the domain structure of WWP1, a Nedd4 family protein. Biochem Biophys Res Commun. 2002;290:431–7. 11779188 10.1006/bbrc.2001.6206

[35] Seo SR, Lallemand F, Ferrand N, Pessah M, L’Hoste S, Camonis J, et al. The novel E3 ubiquitin ligase Tiul1 associates with TGIF to target Smad2 for degradation. EMBO J. 2004;23:3780–92. 15359284 10.1038/sj.emboj.7600398 PMC522797

[36] Lee YR, Chen M, Lee JD, Zhang J, Lin SY, Fu TM, et al. Reactivation of PTEN tumor suppressor for cancer treatment through inhibition of a MYC-WWP1 inhibitory pathway. Science. 2019;364:eaau0159. 10.1126/science.aau0159 PMC7081834 31097636

[37] Ambrozkiewicz MC, Schwark M, Kishimoto-Suga M, Borisova E, Hori K, Salazar-Lazaro A, et al. Polarity Acquisition in Cortical Neurons Is Driven by Synergistic Action of Sox9-Regulated Wwp1 and Wwp2 E3 Ubiquitin Ligases and Intronic miR-140. Neuron. 2018;100:1097–115.e15. 30392800 10.1016/j.neuron.2018.10.008

[38] Bibert S, Quinodoz M, Perriot S, Krebs FS, Jan M, Malta RC, et al. Herpes simplex encephalitis due to a mutation in an E3 ubiquitin ligase. Nat Commun. 2024;15:3969. 38730242 10.1038/s41467-024-48287-0 PMC11087577

[39] Paoli P, Giannoni E, Chiarugi P. Anoikis molecular pathways and its role in cancer progression. Biochim Biophys Acta. 2013;1833:3481–98. 23830918 10.1016/j.bbamcr.2013.06.026

[40] Zhu Z, Fang C, Xu H, Yuan L, Du Y, Ni Y, et al. Anoikis resistance in diffuse glioma: The potential therapeutic targets in the future. Front Oncol. 2022;12:976557. 36046036 10.3389/fonc.2022.976557 PMC9423707

[41] Han YH, Wang Y, Lee SJ, Jin MH, Sun HN, Kwon T. Regulation of anoikis by extrinsic death receptor pathways. Cell Commun Signal. 2023;21:227. 37667281 10.1186/s12964-023-01247-5 PMC10478316

[42] Mei J, Jiang XY, Tian HX, Rong DC, Song JN, Wang L, et al. Anoikis in cell fate, physiopathology, and therapeutic interventions. MedComm. 2024;5:e718. 39286778 10.1002/mco2.718 PMC11401975

[43] Wang X, Li Y, He M, Kong X, Jiang P, Liu X, et al. UbiBrowser 2.0: a comprehensive resource for proteome-wide known and predicted ubiquitin ligase/deubiquitinase-substrate interactions in eukaryotic species. Nucleic Acids Res. 2022;50:D719–28. 34669962 10.1093/nar/gkab962 PMC8728189

[44] Li Y, Cheng Q, Gao J, Chen Z, Guo J, Li Z, et al. WWP1 upregulation predicts poor prognosis and promotes tumor progression by regulating ubiquitination of NDFIP1 in intrahepatic cholangiocarcinoma. Cell Death Discov. 2022;8:107. 35264565 10.1038/s41420-022-00882-0 PMC8906119

[45] Brionne TC, Tesseur I, Masliah E, Wyss-Coray T. Loss of TGF-beta 1 leads to increased neuronal cell death and microgliosis in mouse brain. Neuron. 2003;40:1133–45. 14687548 10.1016/s0896-6273(03)00766-9

[46] Stegmuller J, Huynh MA, Yuan Z, Konishi Y, Bonni A. TGFbeta-Smad2 signaling regulates the Cdh1-APC/SnoN pathway of axonal morphogenesis. J Neurosci. 2008;28:1961–9. 18287512 10.1523/JNEUROSCI.3061-07.2008 PMC6671436

[47] Yi JJ, Barnes AP, Hand R, Polleux F, Ehlers MD. TGF-beta signaling specifies axons during brain development. Cell. 2010;142:144–57. 20603020 10.1016/j.cell.2010.06.010 PMC2933408

[48] Braunger BM, Pielmeier S, Demmer C, Landstorfer V, Kawall D, Abramov N, et al. TGF-beta signaling protects retinal neurons from programmed cell death during the development of the mammalian eye. J Neurosci. 2013;33:14246–58. 23986258 10.1523/JNEUROSCI.0991-13.2013 PMC6618509

[49] Nakashima H, Tsujimura K, Irie K, Ishizu M, Pan M, Kameda T, et al. Canonical TGF-beta Signaling Negatively Regulates Neuronal Morphogenesis through TGIF/Smad Complex-Mediated CRMP2 Suppression. J Neurosci. 2018;38:4791–810. 29695415 10.1523/JNEUROSCI.2423-17.2018 PMC6596019

[50] Rothstein M, Azambuja AP, Kanno TY, Breen C, Simoes-Costa M. TGF-beta signaling controls neural crest developmental plasticity via SMAD2/3. Dev Cell. 2025;60:1686–701. 10.1016/j.devcel.2025.01.018 PMC12187560 39983721

[51] Stipursky J, Francis D, Dezonne RS, Bergamo de Araujo AP, Souza L, Moraes CA, et al. TGF-beta1 promotes cerebral cortex radial glia-astrocyte differentiation in vivo. Front Cell Neurosci. 2014;8:393. 25484855 10.3389/fncel.2014.00393 PMC4240069

[52] Kaneko N, Hirai K, Oshima M, Yura K, Hattori M, Maeda N, et al. ADAMTS2 promotes radial migration by activating TGF-beta signaling in the developing neocortex. EMBO Rep. 2024;25:3090–115. 38871984 10.1038/s44319-024-00174-x PMC11239934

[53] Herbertz S, Sawyer JS, Stauber AJ, Gueorguieva I, Driscoll KE, Estrem ST, et al. Clinical development of galunisertib (LY2157299 monohydrate), a small molecule inhibitor of transforming growth factor-beta signaling pathway. Drug Des Devel Ther. 2015;9:4479–99. 26309397 10.2147/DDDT.S86621 PMC4539082

[54] Schubert M, Klinger B, Klunemann M, Sieber A, Uhlitz F, Sauer S, et al. Perturbation-response genes reveal signaling footprints in cancer gene expression. Nat Commun. 2018;9:20. 29295995 10.1038/s41467-017-02391-6 PMC5750219

[55] Hinz B. The extracellular matrix and transforming growth factor-beta1: Tale of a strained relationship. Matrix Biol. 2015;47:54–65. 25960420 10.1016/j.matbio.2015.05.006

[56] Chakravarthy A, Khan L, Bensler NP, Bose P, De Carvalho DD. TGF-beta-associated extracellular matrix genes link cancer-associated fibroblasts to immune evasion and immunotherapy failure. Nat Commun. 2018;9:4692. 30410077 10.1038/s41467-018-06654-8 PMC6224529

[57] Deng Z, Fan T, Xiao C, Tian H, Zheng Y, Li C, et al. TGF-beta signaling in health, disease, and therapeutics. Signal Transduct Target Ther. 2024;9:61. 38514615 10.1038/s41392-024-01764-w PMC10958066

[58] Moren A, Imamura T, Miyazono K, Heldin CH, Moustakas A. Degradation of the tumor suppressor Smad4 by WW and HECT domain ubiquitin ligases. J Biol Chem. 2005;280:22115–23. 15817471 10.1074/jbc.M414027200

[59] Kano J, Wang H, Zhang H, Noguchi M. Roles of DKK3 in cellular adhesion, motility, and invasion through extracellular interaction with TGFBI. FEBS J. 2022;289:6385–99. 35574828 10.1111/febs.16529

[60] Fournier PG, Juarez P, Jiang G, Clines GA, Niewolna M, Kim HS, et al. The TGF-beta Signaling Regulator PMEPA1 Suppresses Prostate Cancer Metastases to Bone. Cancer Cell. 2015;27:809–21. 25982816 10.1016/j.ccell.2015.04.009 PMC4464909

[61] Huang S, Wa Q, Pan J, Peng X, Ren D, Li Q, et al. Transcriptional downregulation of miR-133b by REST promotes prostate cancer metastasis to bone via activating TGF-beta signaling. Cell Death Dis. 2018;9:779. 30006541 10.1038/s41419-018-0807-3 PMC6045651

[62] Cao Y, Agarwal R, Dituri F, Lupo L, Trerotoli P, Mancarella S, et al. NGS-based transcriptome profiling reveals biomarkers for companion diagnostics of the TGF-beta receptor blocker galunisertib in HCC. Cell Death Dis. 2017;8:e2634. 28230858 10.1038/cddis.2017.44 PMC5386488

[63] Verdecia MA, Joazeiro CA, Wells NJ, Ferrer JL, Bowman ME, Hunter T, et al. Conformational flexibility underlies ubiquitin ligation mediated by the WWP1 HECT domain E3 ligase. Mol Cell. 2003;11:249–59. 12535537 10.1016/s1097-2765(02)00774-8

[64] Schymkowitz J, Borg J, Stricher F, Nys R, Rousseau F, Serrano L. The FoldX web server: an online force field. Nucleic Acids Res. 2005;33:W382–8. 15980494 10.1093/nar/gki387 PMC1160148

[65] Wu Z, Zan P, Li S, Liu J, Wang J, Chen D, et al. Knockdown of WWP1 inhibits growth and invasion, but induces apoptosis of osteosarcoma cells. Int J Clin Exp Pathol. 2015;8:7869–77. 26339351 PMC4555679

[66] Tripathi S, Levine H, Jolly MK. The Physics of Cellular Decision Making During Epithelial-Mesenchymal Transition. Annu Rev Biophys. 2020;49:1–18. 31913665 10.1146/annurev-biophys-121219-081557

[67] Frisch SM, Schaller M, Cieply B. Mechanisms that link the oncogenic epithelial-mesenchymal transition to suppression of anoikis. J Cell Sci. 2013;126:21–9. 23516327 10.1242/jcs.120907 PMC3603508

[68] Dai Y, Zhang X, Ou Y, Zou L, Zhang D, Yang Q, et al. Anoikis resistance-protagonists of breast cancer cells survive and metastasize after ECM detachment. Cell Commun Signal. 2023;21:190. 37537585 10.1186/s12964-023-01183-4 PMC10399053

[69] Weems AD, Welf ES, Driscoll MK, Zhou FY, Mazloom-Farsibaf H, Chang BJ, et al. Blebs promote cell survival by assembling oncogenic signalling hubs. Nature. 2023;615:517–25. 36859545 10.1038/s41586-023-05758-6 PMC10881276

[70] Insolera R, Bazzi H, Shao W, Anderson KV, Shi SH. Cortical neurogenesis in the absence of centrioles. Nat Neurosci. 2014;17:1528–35. 25282615 10.1038/nn.3831 PMC4213237

[71] Kielar M, Tuy FP, Bizzotto S, Lebrand C, de Juan Romero C, Poirier K, et al. Mutations in Eml1 lead to ectopic progenitors and neuronal heterotopia in mouse and human. Nat Neurosci. 2014;17:923–33. 24859200 10.1038/nn.3729

[72] Ferland RJ, Batiz LF, Neal J, Lian G, Bundock E, Lu J, et al. Disruption of neural progenitors along the ventricular and subventricular zones in periventricular heterotopia. Hum Mol Genet. 2009;18:497–516. 18996916 10.1093/hmg/ddn377 PMC2722192

[73] Chen H, Moreno-Moral A, Pesce F, Devapragash N, Mancini M, Heng EL, et al. WWP2 regulates pathological cardiac fibrosis by modulating SMAD2 signaling. Nat Commun. 2019;10:3616. 31399586 10.1038/s41467-019-11551-9 PMC6689010

[74] Li Y, Zhou Z, Chen C. WW domain-containing E3 ubiquitin protein ligase 1 targets p63 transcription factor for ubiquitin-mediated proteasomal degradation and regulates apoptosis. Cell Death Differ. 2008;15:1941–51. 18806757 10.1038/cdd.2008.134

[75] Novelli G, Liu J, Biancolella M, Alonzi T, Novelli A, Patten JJ, et al. Inhibition of HECT E3 ligases as potential therapy for COVID-19. Cell Death Dis. 2021;12:310. 33762578 10.1038/s41419-021-03513-1 PMC7987752

[76] Tucker WO, Kinghorn AB, Fraser LA, Cheung YW, Tanner JA. Selection and characterization of a DNA aptamer specifically targeting human HECT ubiquitin ligase WWP1. Int J Mol Sci. 2018;19:763. 10.3390/ijms19030763 PMC5877624 29518962

[77] Fan H, Hu X, Cao F, Zhou L, Wen R, Shen H, et al. WWP1 inhibition increases SHP2 inhibitor efficacy in colorectal cancer. NPJ Precis Oncol. 2024;8:144. 39014007 10.1038/s41698-024-00650-6 PMC11252267

[78] Dudey AP, Hughes GR, Rigby JM, Monaco S, Stephenson GR, Storr TE, et al. 3,3’-Diindolylmethane (DIM): A Molecular Scaffold for Inhibition of WWP1 and WWP2, Members of the NEDD4 Family HECT E3 Ligases. ACS Omega. 2025;10:5963–72. 39989805 10.1021/acsomega.4c09944 PMC11840788

[79] Mitsuhashi N, Toyo-Oka L, Katayama T, Kawashima M, Kawashima S, Miyazaki K, et al. TogoVar: a comprehensive Japanese genetic variation database. Hum Genome Var. 2022;9:44. 36509753 10.1038/s41439-022-00222-9 PMC9744889

[80] Karczewski KJ, Francioli LC, Tiao G, Cummings BB, Alfoldi J, Wang Q, et al. The mutational constraint spectrum quantified from variation in 141,456 humans. Nature. 2020;581:434–43. 32461654 10.1038/s41586-020-2308-7 PMC7334197

[81] Rosanoff MJ, Ottman R. Penetrance of LGI1 mutations in autosomal dominant partial epilepsy with auditory features. Neurology. 2008;71:567–71. 18711109 10.1212/01.wnl.0000323926.77565.ee PMC2652575

[82] Chen C, Fang F, Wang X, Lv J, Wang X, Jin H. Phenotypic and Genotypic Characteristics of SCN1A Associated Seizure Diseases. Front Mol Neurosci. 2022;15:821012. 35571373 10.3389/fnmol.2022.821012 PMC9096348

[83] Goret M, Edelweiss E, Jehl J, Reiss D, Aguirre-Pineda P, Friant S, et al. Combining dynamin 2 myopathy and neuropathy mutations rescues both phenotypes. Nat Commun. 2025;16:4667. 40393994 10.1038/s41467-025-59925-6 PMC12092598

[84] Arboleda-Velasquez JF, Lopera F, O’Hare M, Delgado-Tirado S, Marino C, Chmielewska N, et al. Resistance to autosomal dominant Alzheimer’s disease in an APOE3 Christchurch homozygote: a case report. Nat Med. 2019;25:1680–3. 31686034 10.1038/s41591-019-0611-3 PMC6898984

[85] Kim YE, Kim YS, Lee HE, So KH, Choe Y, Suh BC, et al. Reversibility and developmental neuropathology of linear nevus sebaceous syndrome caused by dysregulation of the RAS pathway. Cell Rep. 2023;42:112003. 36641749 10.1016/j.celrep.2023.112003

[86] Subramanian A, Tamayo P, Mootha VK, Mukherjee S, Ebert BL, Gillette MA, et al. Gene set enrichment analysis: a knowledge-based approach for interpreting genome-wide expression profiles. Proc Natl Acad Sci USA. 2005;102:15545–50. 16199517 10.1073/pnas.0506580102 PMC1239896

[87] Van der Auwera GA, Carneiro MO, Hartl C, Poplin R, Del Angel G, Levy-Moonshine A, et al. From FastQ data to high confidence variant calls: the Genome Analysis Toolkit best practices pipeline. Curr Protoc Bioinforma. 2013;43:11 0 1–0 33. 10.1002/0471250953.bi1110s43 PMC4243306 25431634

