# Supplementary material for: WWP1 gain-of-function drives developmental anoikis through TGFβ pathway during neurodevelopment
Source: Cell Death Discov. 2026 Mar 6;12:133. doi: 10.1038/s41420-026-02977-4 (PMC13039840; doi:10.1038/s41420-026-02977-4)
Supplement: Supplementary file 1 — Supplementary Information [file 41420_2026_2977_MOESM1_ESM.docx]

**Supplementary Information**

**WWP1 gain-of-function drives developmental anoikis through TGFβ pathway during neurodevelopment**

Authors: Ki Hurn So^1^*, Seungbok Lee^2,3^*, Jiayi Wong^1^*, Hyunsik Lee^4,5^, Eun-Jin Yun^1^, Se Song Jang^3^, Hee-Jung Choi^4^, Jong-Hee Chae^2,3^, Seung Tae Baek^1^

Affiliations:

^1^Department of Life Sciences, Pohang University of Science and Technology (POSTECH), Pohang 37673, Republic of Korea.

^2^Department of Genomic Medicine, Seoul National University Hospital, Seoul 03080, Republic of Korea.

^3^Department of Pediatrics, Seoul National University College of Medicine, Seoul 03080, Republic of Korea.

^4^Department of Biological Sciences, Seoul National University, Seoul 08826, Republic of Korea.

^5^Bio R&D Center, Samsung Biologics, Incheon, South Korea

*These authors contributed equally to this work.

Corresponding author:

Jong-Hee Chae, M.D., Ph.D. Email: chaeped1@snu.ac.kr

Seung Tae Baek, Ph.D. Email: sbaek@postech.ac.kr

**This PDF file includes:**

**Supplemental Figures 1 to 6 and their legends**

**Table S1. Primers used in this study**

**Legends for Movie S1 and S2**

**
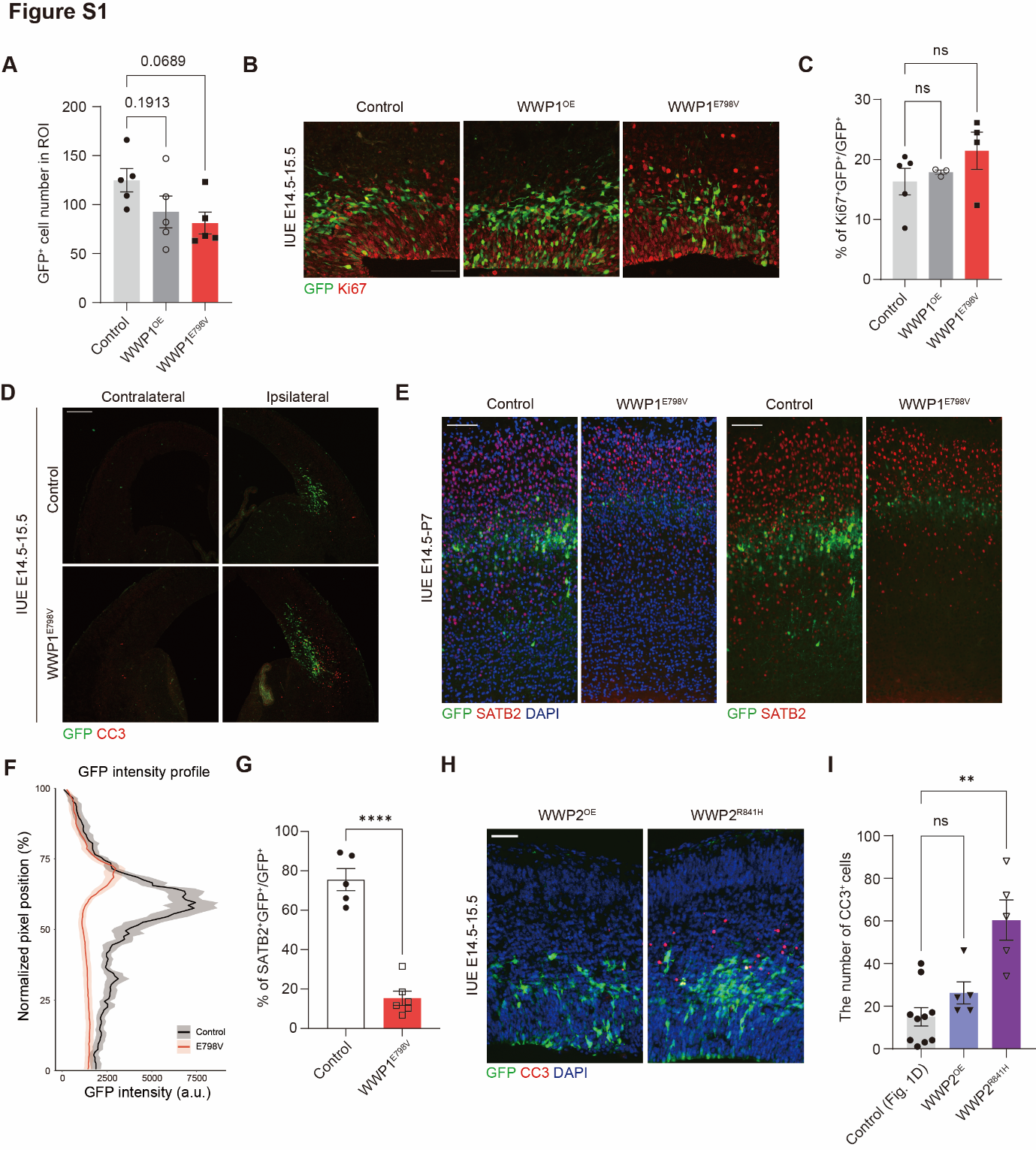
**

**Fig S1. WWP1 GOF-induced neurodevelopmental defects.**

**A.** Quantification of total GFP^+^ cells in E18.5 brain sections electroporated with control, WWP1^OE^, and WWP1^E798V^. *n* = 5 per group. One-way ANOVA with Dunnett’s post hoc test. *P* values are indicated. **B.** Representative images of E15.5 brain sections electroporated with control or WWP1 variants. Green, GFP; red, Ki67. Scale bar, 50 μm. **C.** Quantification of Ki67^+^ cells among GFP^+^ cells in the electroporated region. Control, *n* = 5; WWP1^OE^, *n* = 3; WWP1^E798V^, *n* = 4. Kruskal-Wallis test with Dunn’s post hoc test. ns, not significant. **D.** Representative images ipsilateral and contralateral sides of E15.5 brain sections electroporated with control or WWP1^E798V^. Green, GFP; red, CC3. Scale bar, 200 μm. **E.** Representative images of P7 brain sections electroporated with control and WWP1^E798V^ constructs. Green, GFP; red, SATB2; blue, DAPI. Scale bar, 100 μm. **F.** Quantification of GFP intensity across P7 ipsilateral cortical plates electroporated with control and WWP1^E798V^ constructs. Black, control, *n* = 5; red, WWP1^E798V^, *n* = 6. The bold solid lines indicate the mean GFP intensity, and the shaded areas represent the SEM. **G.** Quantification of SATB2^+^ among GFP^+^ cells in P7 brains electroporated with control and WWP1^E798V^ constructs. Control, *n* = 5; WWP1^E798V^, *n* = 6. Student’s two-tailed t-test. *****p* < 0.0001. **H.** Representative images of E15.5 brain sections electroporated with WWP2^OE^ or WWP2^R841H^. Green, GFP; red, CC3; blue, DAPI. Scale bar, 50 μm. **I.** Quantification of total CC3^+^ in the electroporated region. Control data is from Fig. 1D. WWP2^OE^, *n* = 5; WWP2^R841H^, *n* = 5. Kruskal-Wallis test with Dunn’s post hoc test. ***p* < 0.01; ns, not significant. Bar graphs indicate mean ± SEM.


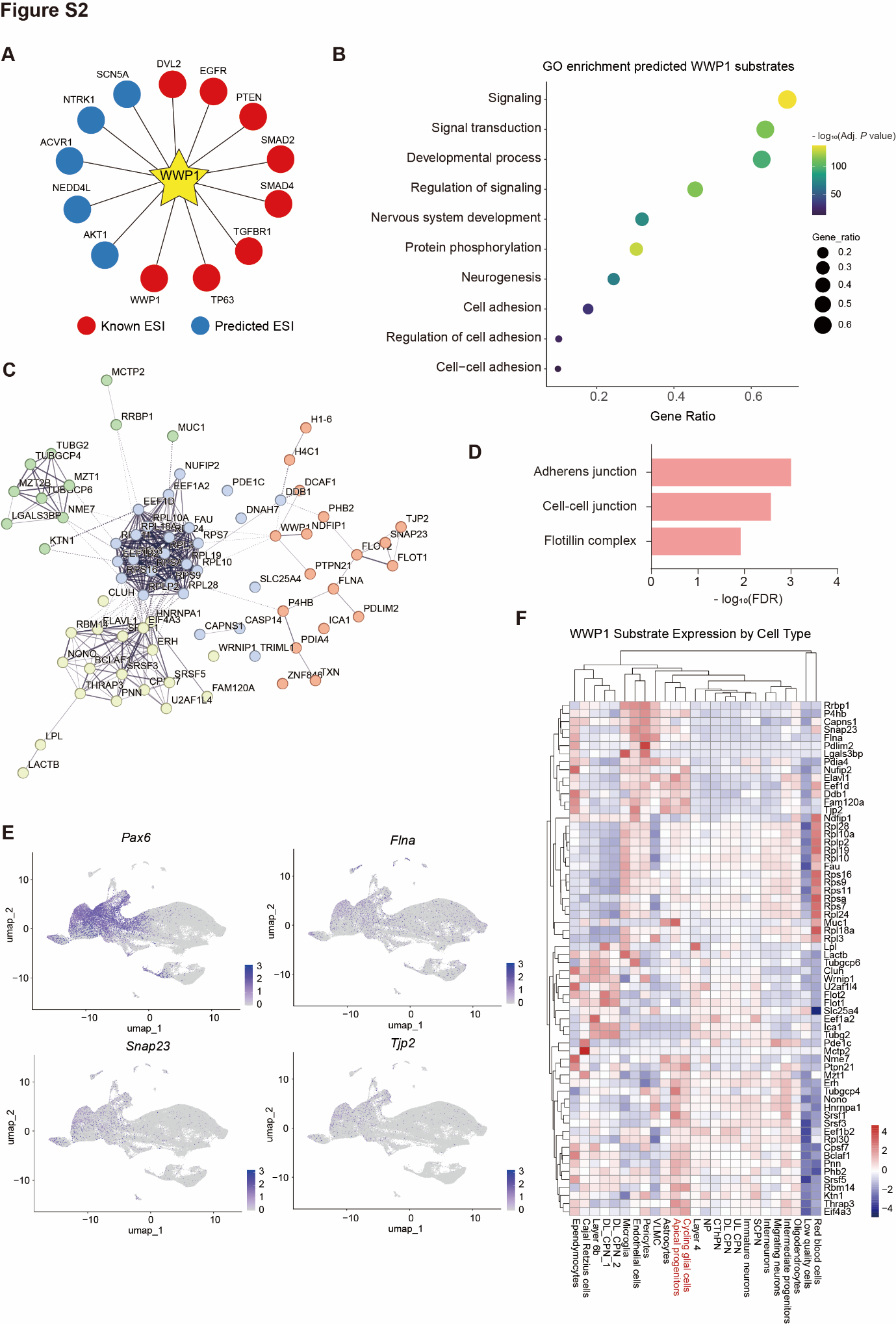


**Fig S2. Adhesion-related WWP1 substrates enriched neurodevelopment.**

**A.** Known and predicted WWP1 substrates derived from the Ubibrowser 2.0 database. ESI, enzyme-substrate interaction. **B.** Functional gene ontology enrichment analysis of predicted WWP1 substrates. Color indicates negative log base 10 of adjusted *P* value, and circle size indicates gene ratio. **C.** Protein-protein interaction network of 76 WWP1-interacting proteins constructed from the STRING database. Four groups are clustered by k-means clustering. **D.** Functional gene ontology enrichment analysis of one of the WWP1 substrate clusters. **E.** Visualization of expression distribution of adhesion-related WWP1-interacting protein-encoding genes (*Flna*, *Snap23*, *Tjp2*) during mouse prenatal neurodevelopment. *Pax6* is used to visualize the apical progenitor cell population. **F.** Heatmap of WWP1-interacting protein-encoding gene expression in different cell types in the developing mouse brain. Apical progenitors and cycling glial cells are indicated in red. Single-cell data was re-analyzed from GSE153164 in **E** and **F**.


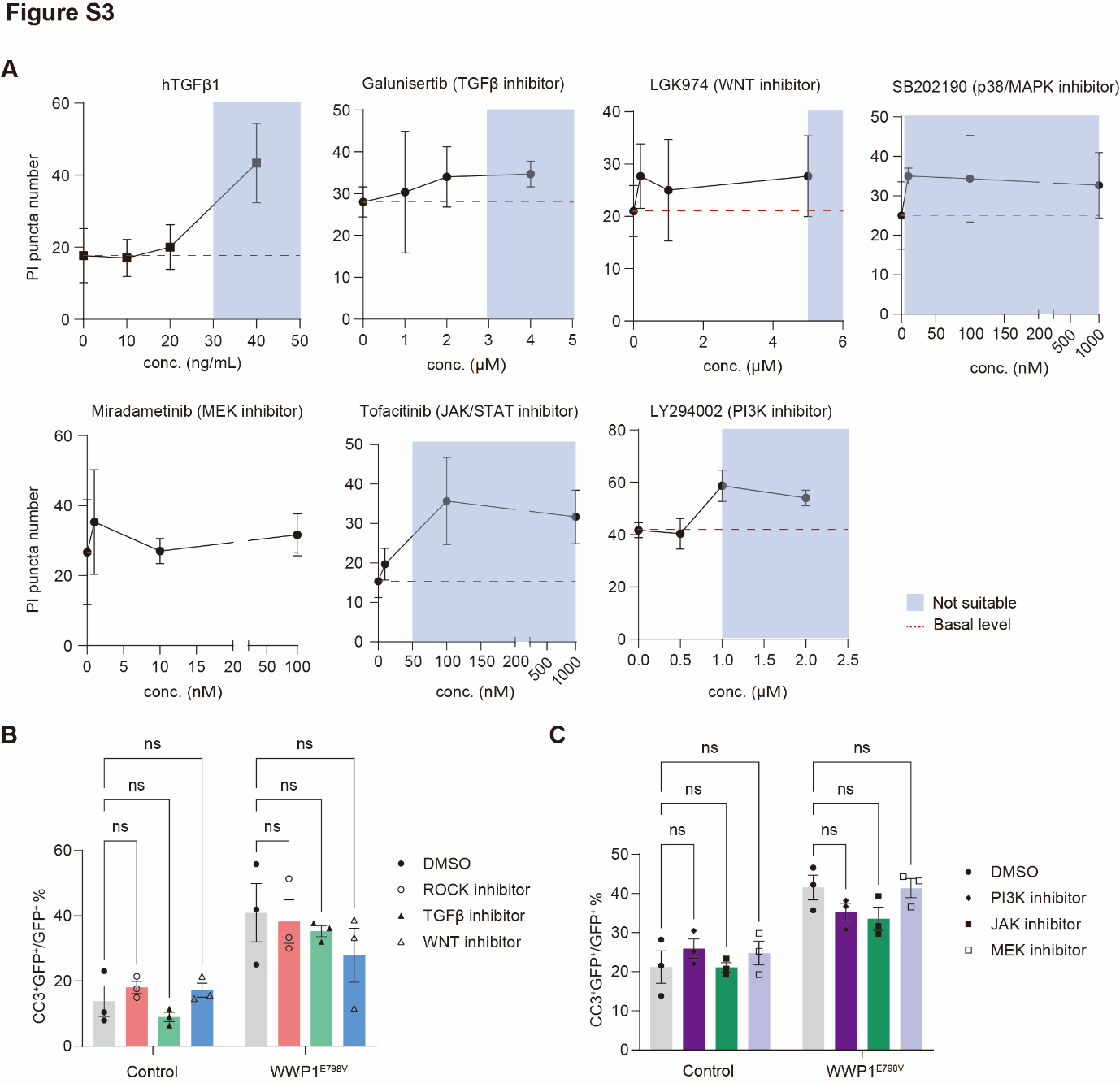


**Fig S3. Pathway-oriented screening of WWP1 GOF-induced cell death.**

**A.** Quantification of dead NPC count indicated by propidium iodide treated with pathway ligand or inhibitors. The red dashed line indicates the basal cell death level, and the blue box area indicates toxic concentrations. **B.** Quantification of the percentage of apoptotic cells among GFP^+^ cells transfected with control or WWP1^E798V^. Cells were treated with DMSO, ROCK, TGFβ, or WNT inhibitors. *n* = 3. Two-way ANOVA with Dunnett’s post hoc test. ns, not significant. **C.** Quantification of the percentage of apoptotic cells among GFP^+^ cells transfected with control or WWP1^E798V^. Cells were treated with DMSO, PI3K, JAK, or MEK inhibitors. *n* = 3. Two-way ANOVA with Dunnett’s post hoc test. ns, not significant. Bar graphs indicate mean ± SEM.


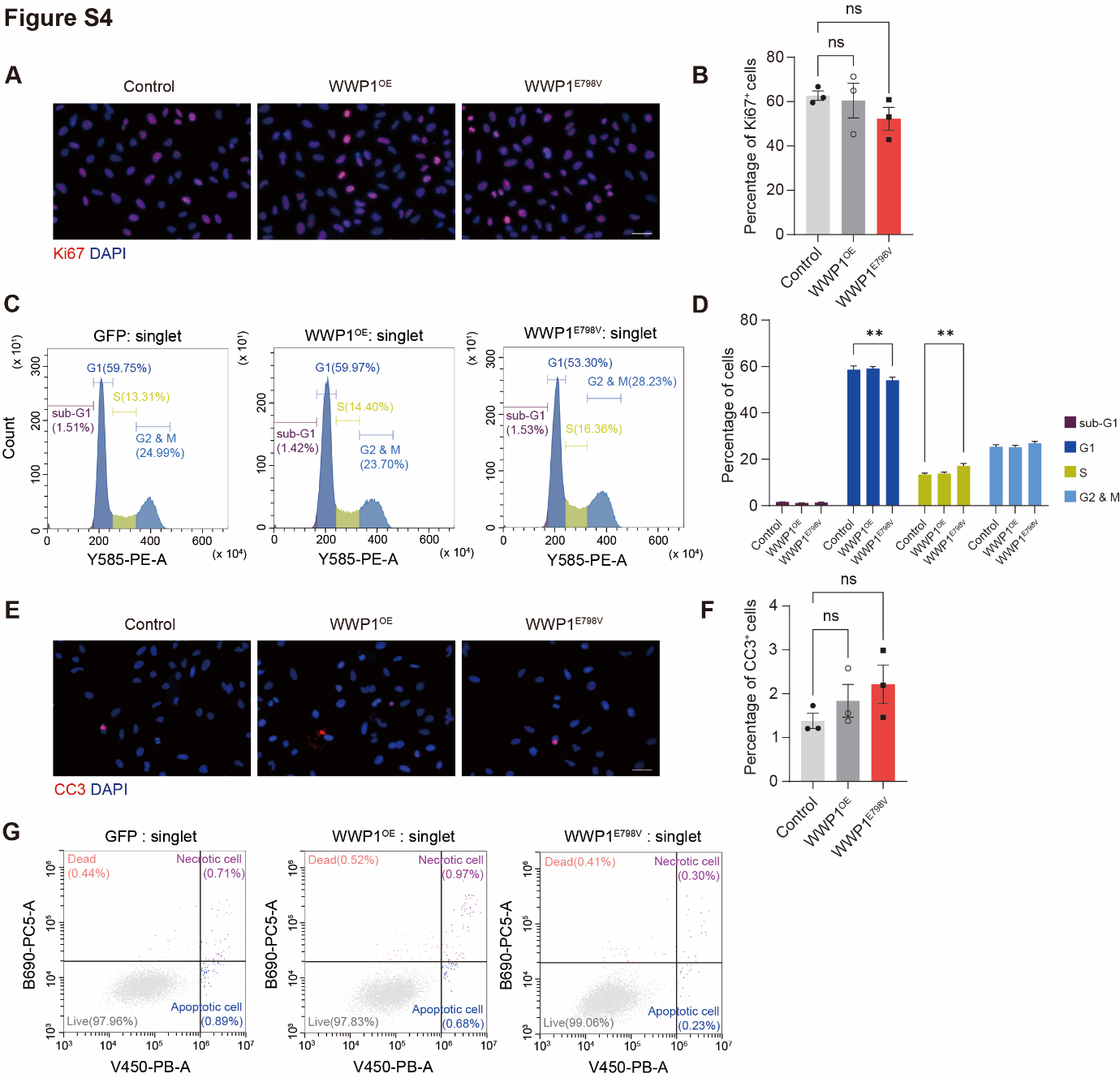


**Fig S4. Basal cellular characterization in WWP1 HeLa cell lines.**

**A.** Representative images of control, WWP1^OE^, or WWP1^E798V^ HeLa cell lines stained with Ki67. Red, Ki67; blue, DAPI. Scale bar, 100 μm. **B.** Quantification of the percentages of Ki67^+^ cells in WWP1 cell lines. *n* = 3 for each condition. Kruskal-Wallis test with Dunn’s post hoc test. ns, not significant. **C.** Representative images of cell cycle analysis in control, WWP1^OE^, or WWP1^E798V^ HeLa cell lines using flow cytometry. **D.** Quantification of the percentages of cells in each cell cycle phase in WWP1 cell lines. *n* = 3 for each condition. Two-way ANOVA with Tukey’s post hoc test. ***p* < 0.01. **E.** Representative images of control, WWP1^OE^, or WWP1^E798V^ HeLa cell lines stained with CC3. Red, CC3; blue, DAPI. Scale bar, 100 μm. **F.** Quantification of the percentages of CC3^+^ cells in WWP1 cell lines. *n* = 3 for each condition. Kruskal-Wallis test with Dunn’s post hoc test. ns, not significant. **G.** Representative flow cytometry plots showing Annexin V and 7-AAD intensity in control, WWP1^OE^, and WWP1^E798V^ HeLa cell lines. Cells are categorized into live, early apoptotic, late apoptotic, and necrotic based on their staining patterns. Grey: live; blue: early apoptotic; purple: late apoptotic; red: necrotic. Bar graphs indicate mean ± SEM.


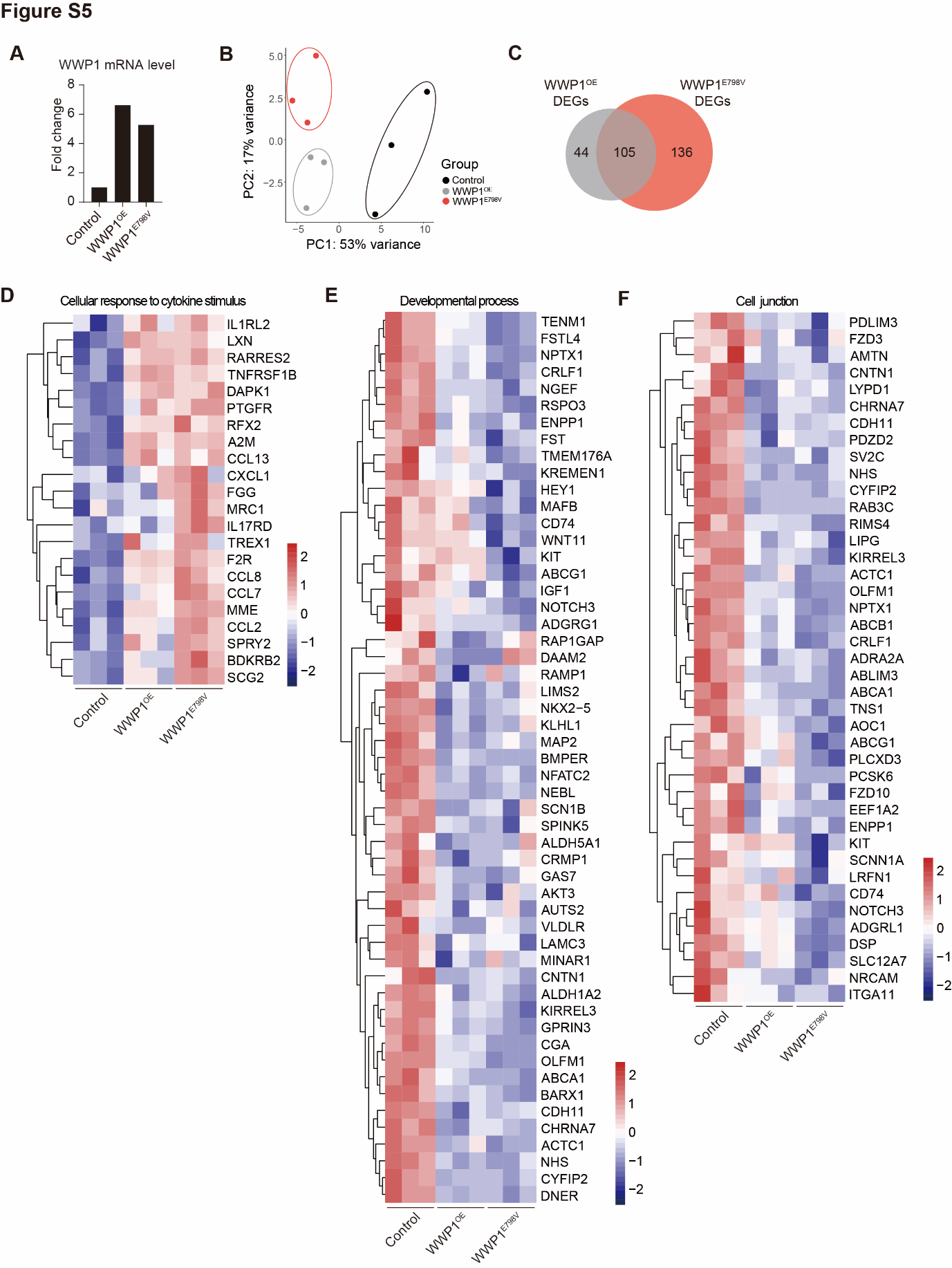


**Fig S5. Transcriptomic analysis of WWP1 HeLa cell lines.**

**A.** Bar plot showing average fold change of *WWP1* mRNA expression level in WWP1^OE^ and WWP1^E798V^ cell lines. **B.** Principal component analysis of RNA sequencing samples. Each color indicates different experimental conditions. **C.** Venn diagram showing overlap between WWP1^OE^ and WWP1^E798V^ DEGs, each compared with the control. **D-F.** Heatmaps of gene expression related to **D)** cellular response to cytokine stimulus, **E)** developmental process, and **F)** cell junction. Target gene lists were from gprofiler gene ontology analysis, and the color gradient represents *Z*-scores.
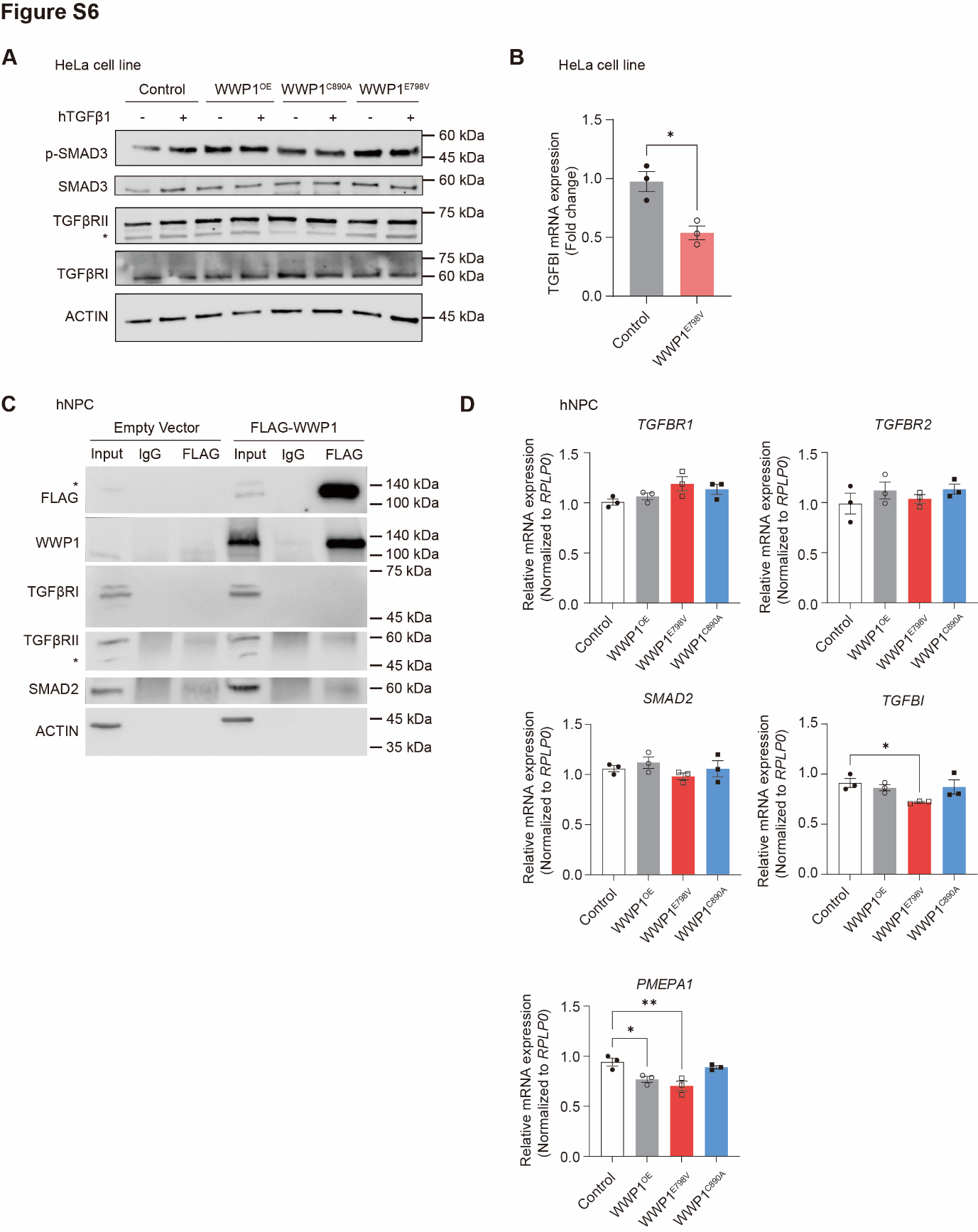


**Fig S6. The impact of WWP1 GOF on TGFβ pathway components at the protein and mRNA levels.**

**A.** Western blot analysis of signaling components in the TGFβ pathway in WWP1 cell lines with or without hTGFβ1 (10 ng/mL) treatment. ACTIN served as a loading control. Asterisk indicates non-specific bands. **B.** The expression level of *TGFBI* mRNA was analyzed by qPCR, normalized to *RPLP0*. *n* = 3. Student’s two-tailed t-test, **p* < 0.05. Bar graphs indicate mean ± SEM. **C.** Coimmunoprecipitation of TGFβ pathway components in hNPCs were transfected with empty vector or FLAG-WWP1. Cell lysates were subjected to immunoprecipitation with anti-FLAG antibody or control IgG, followed by immunoblotting with the indicated antibodies. Input lanes represent 10% of the total lysate before immunoprecipitation. ACTIN served as a loading control. Asterisk indicates non-specific bands. **D.** The expression level of *TGFBR1*, *TGFBR2*, *SMAD2*, *TGFBI*, and *PMEPA1* mRNA normalized to *RPLP0* in hNPCs transfected with control or WWP1 variants. *n* = 3. One-way ANOVA with Dunnett’s post hoc test. **p* < 0.05; ***p* < 0.01. Bar graphs indicate mean ± SEM.

**Table S1. Primers used in this study**

|  | Forward (5'-3') | Reverse (5'-3') |
| --- | --- | --- |
| Human WWP1 cDNA cloning primers | AAAGATATCGCCACCATGGCCACTGCTTCACCAAGGTC | AAAGAATTCTCATTCTTGTCCAAATCCCTCTGTCTCTTCT |
| p.D793N mutagenesis primer | GGCTACAGTACTTCAATGAAAAAGAATTAGAGG | CCTCTAATTCTTTTTCATTGAAGTACTGTAGCC |
| p.E798V mutagenesis primer | GCTACAGTACTTCGATGAAAAAGTATTAGAGGTTATGTTGT | ACAACATAACCTCTAATACTTTTTCATCGAAGTACTGTAGC |
| p.C890A mutagenesis primer | CCAAGAAGCCATACAGCTTTTAATCGCTTG | CAAGCGATTAAAAGCTGTATGGCTTCTTGG |
| WWP1 variants subcloning into pLV-EF1a-MCS-GFP | AAACCTGCAGGGCCACCATGGACTACAAG | AAAGGTATCGATGTCGACTCATTCTTGTCCAAATCCC |
| Human WWP2 cDNA cloning primers | AAAGATATCGCCACCATGGCATCTGCCAGCTCTAGC | AAAGAATTCTTACTCCTGTCCAAAGCCCTCG |
| p.R841H mutagenesis primer | CTGCTTCAACCATCTGGATCTTC | TTTAAGCTTCCAGAGGAACTGCTTCCTTCAC |
| Human *TGFBR1* qPCR primer | ACCGCACTGTCATTCACCAT | ACAGCAACTTCTTCTCCCCG |
| Human *TGFBR2* qPCR primer | TAGGACTGCCCATCCACTGA | TTGGGGTCATGGCAAACTGT |
| Human *SMAD2* qPCR primer | GCTGGCCTGATCTTCACAGT | CCAGAGGCGGAAGTTCTGTT |
| Human *TGFBI* qPCR primer | AGGCCTTCGAGAAGATCCCT | GAGATGATCGCCTTCCCGTT |
| Human *PMEPA1* qPCR primer | GTGCAACTGCAAACGCTCTT | ATGAAGGACCGTGCAGACAG |
| Human *RPLP0* qPCR primer | GGCACCATTGAAATCCTGAG | GACCAGCCCAAAGGAGAAG |

**Movie Legends**

S1. Living imaging of human NPC expressing GFP and treated with CC3-dependent fluorescent dye for cell fate tracking.

S2. Living imaging of human NPC expressing WWP1^E798V^ and treated with CC3-dependent fluorescent dye for cell fate tracking.
