# Supplementary figures and images for: WWP1 gain-of-function drives developmental anoikis through TGFβ pathway during neurodevelopment
Source: Cell Death Discov. 2026 Mar 6;12:133. doi: 10.1038/s41420-026-02977-4 (PMC13039840; doi:10.1038/s41420-026-02977-4)

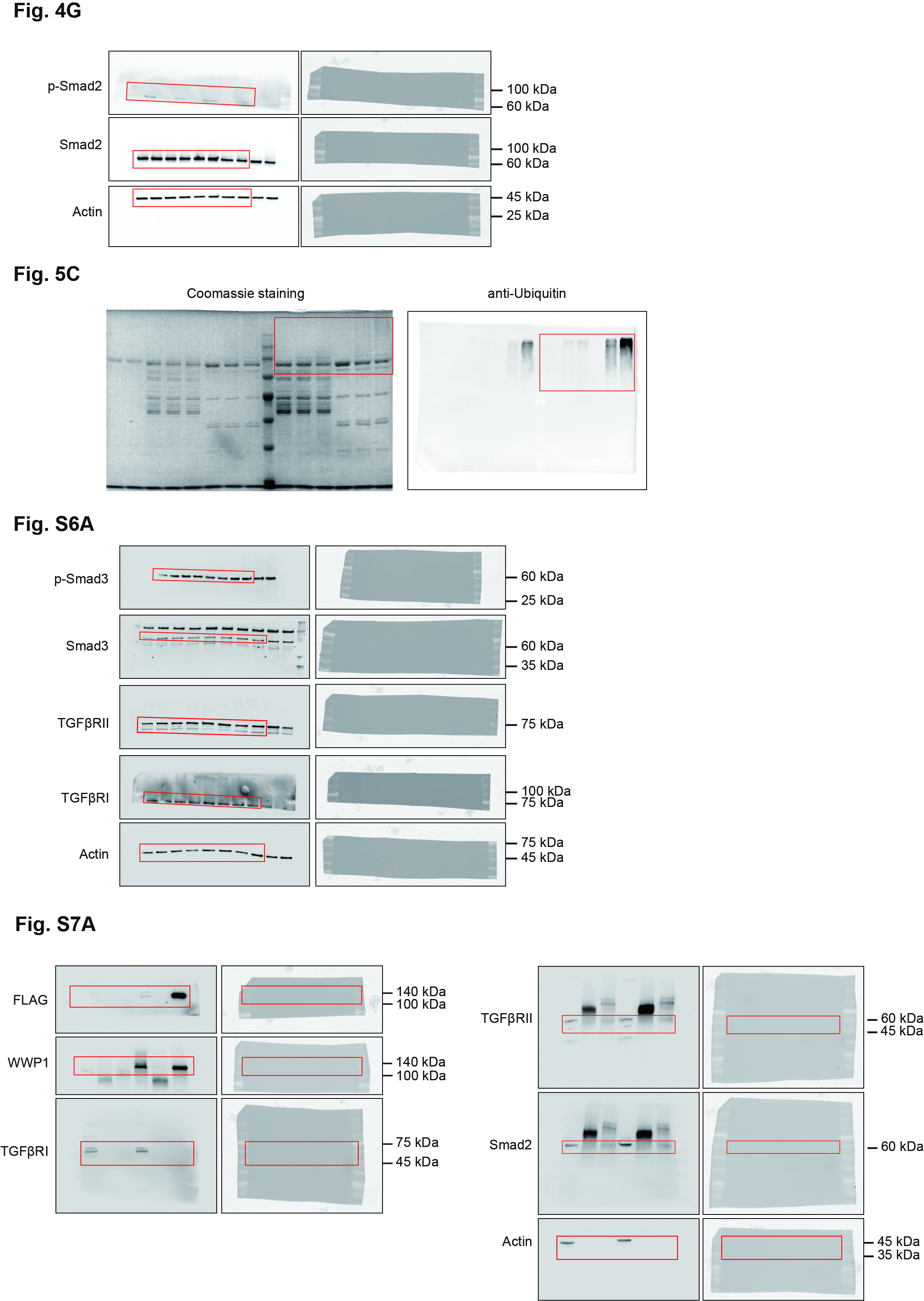

Supplement: Supplementary file 2 — Original Western Blot image [file 41420_2026_2977_MOESM2_ESM.tif]
